# Supplementary figures and images for: Translatome analysis reveals cellular network in DLK-dependent hippocampal glutamatergic neuron degeneration
Source: eLife. 2025 Mar 11;13:RP101173. doi: 10.7554/eLife.101173 (PMC11896613; doi:10.7554/eLife.101173)

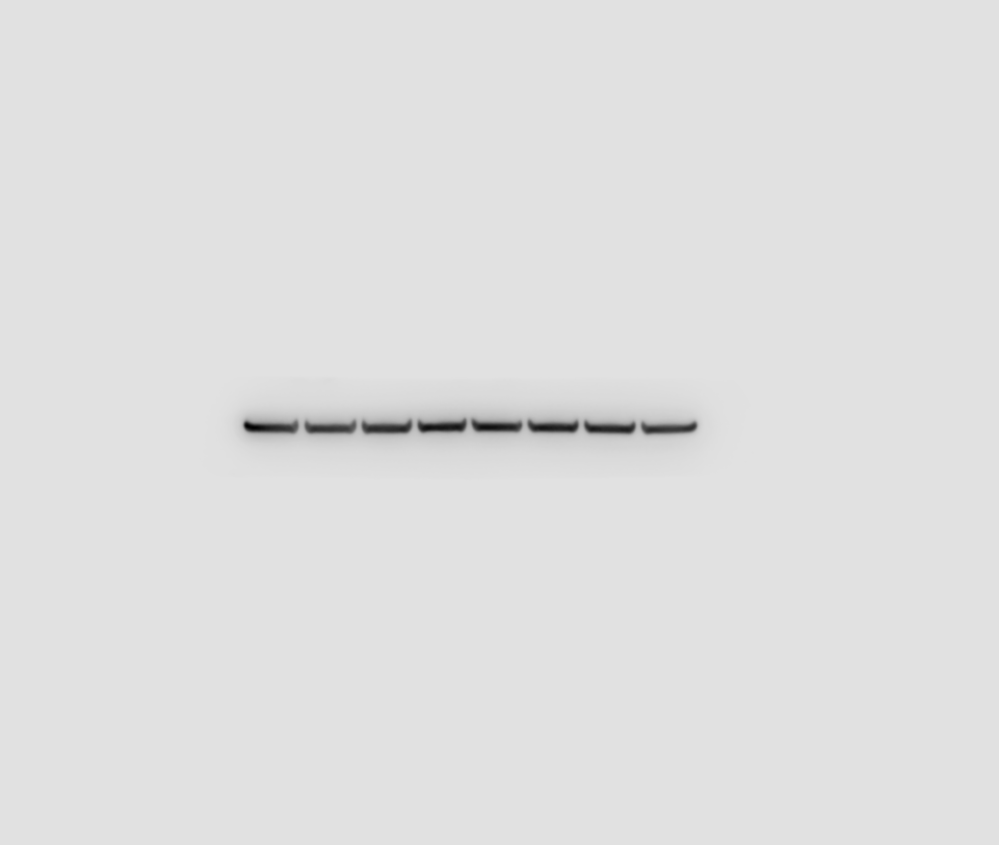

Supplement: Figure 1—source data 1. [file elife-101173-fig1-data1.zip › Figure 1-source data 1/063023_WB_beta-actin_0.5 min_membraneA.tif]

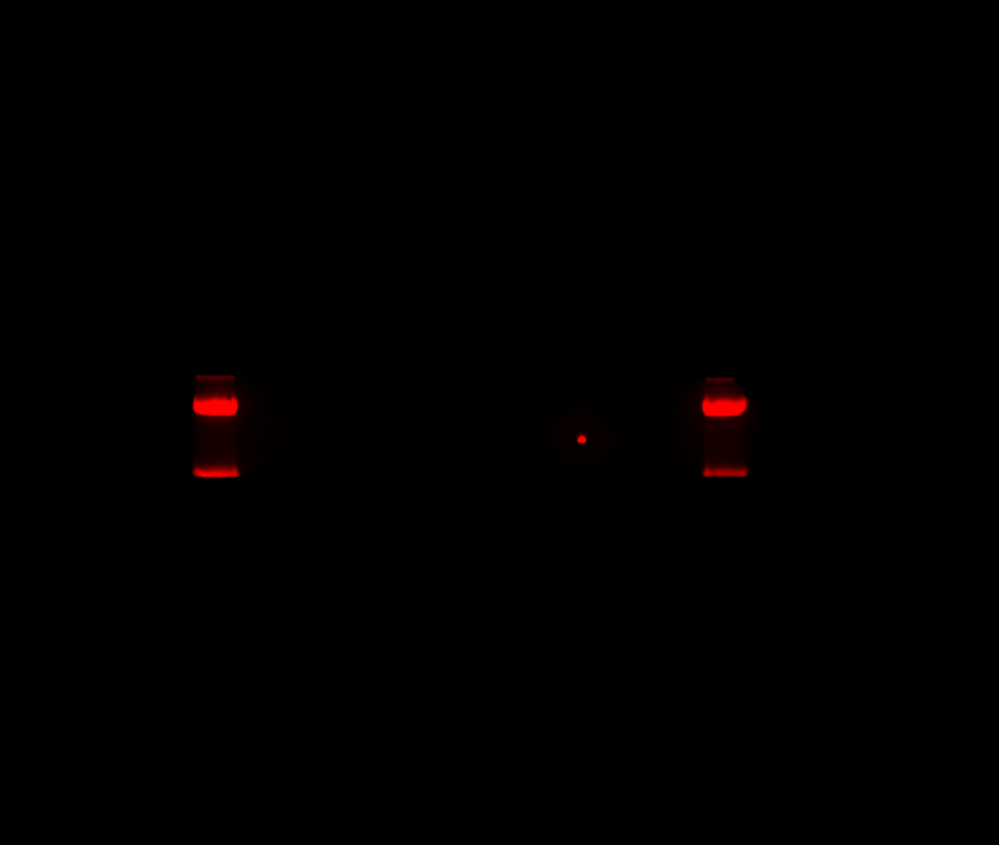

Supplement: Figure 1—source data 1. [file elife-101173-fig1-data1.zip › Figure 1-source data 1/063023_WB_beta-actin_ladder_membraneA.tif]

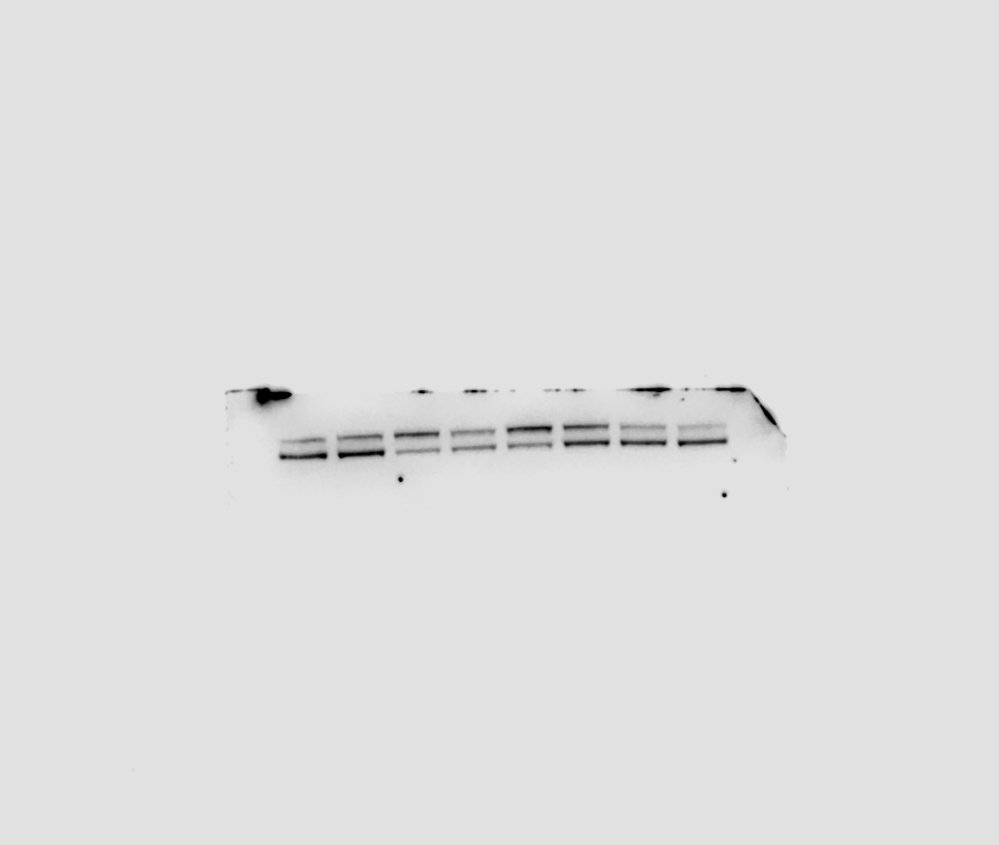

Supplement: Figure 1—source data 1. [file elife-101173-fig1-data1.zip › Figure 1-source data 1/063023_WB_DLK_membrane A_2 min.tif]

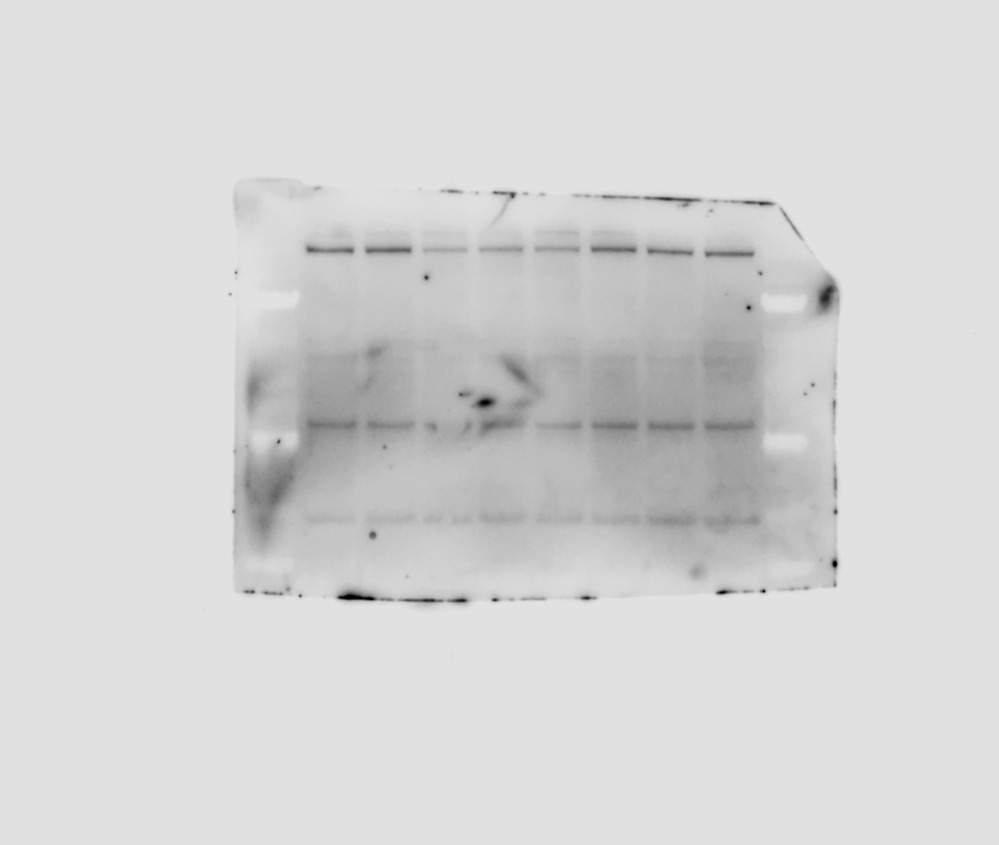

Supplement: Figure 1—figure supplement 1—source data 1. [file elife-101173-fig1-figsupp1-data1.zip › Figure 1-figure supplement 1-source data 1/062923_WB_DLK_10 min_membraneA.tif]

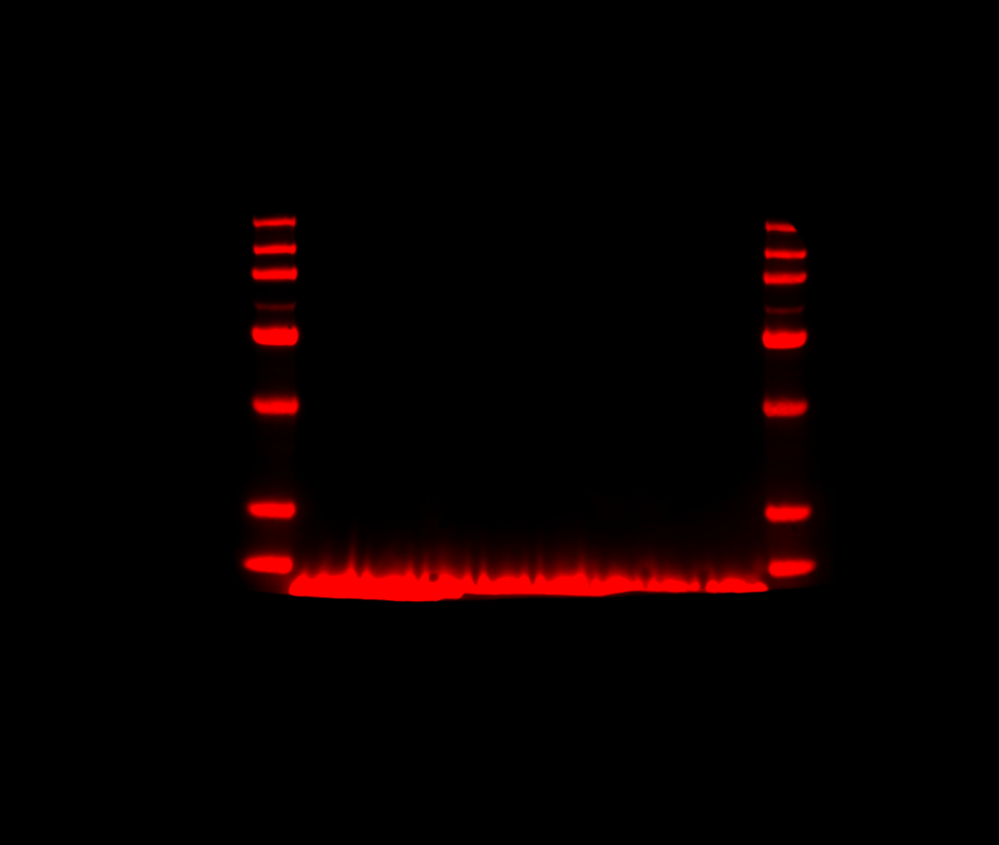

Supplement: Figure 1—figure supplement 1—source data 1. [file elife-101173-fig1-figsupp1-data1.zip › Figure 1-figure supplement 1-source data 1/062923_WB_DLK_ladder_membraneA.tif]

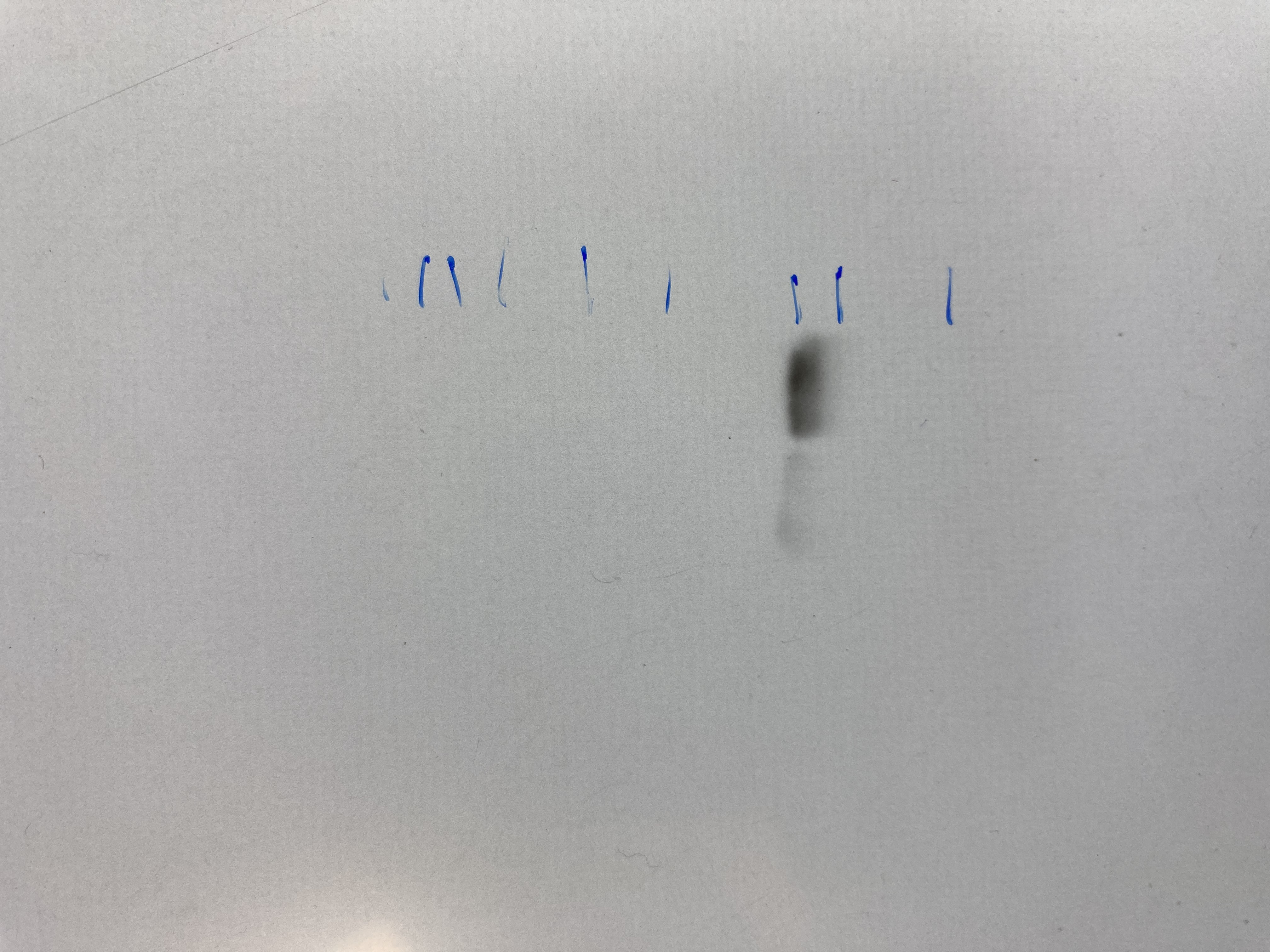

Supplement: Figure 3—figure supplement 1—source data 1. [file elife-101173-fig3-figsupp1-data1.zip › Figure 3-figure supplement 1-source data 1/IP HA tag of DRV43 good 10min expose.jpg]

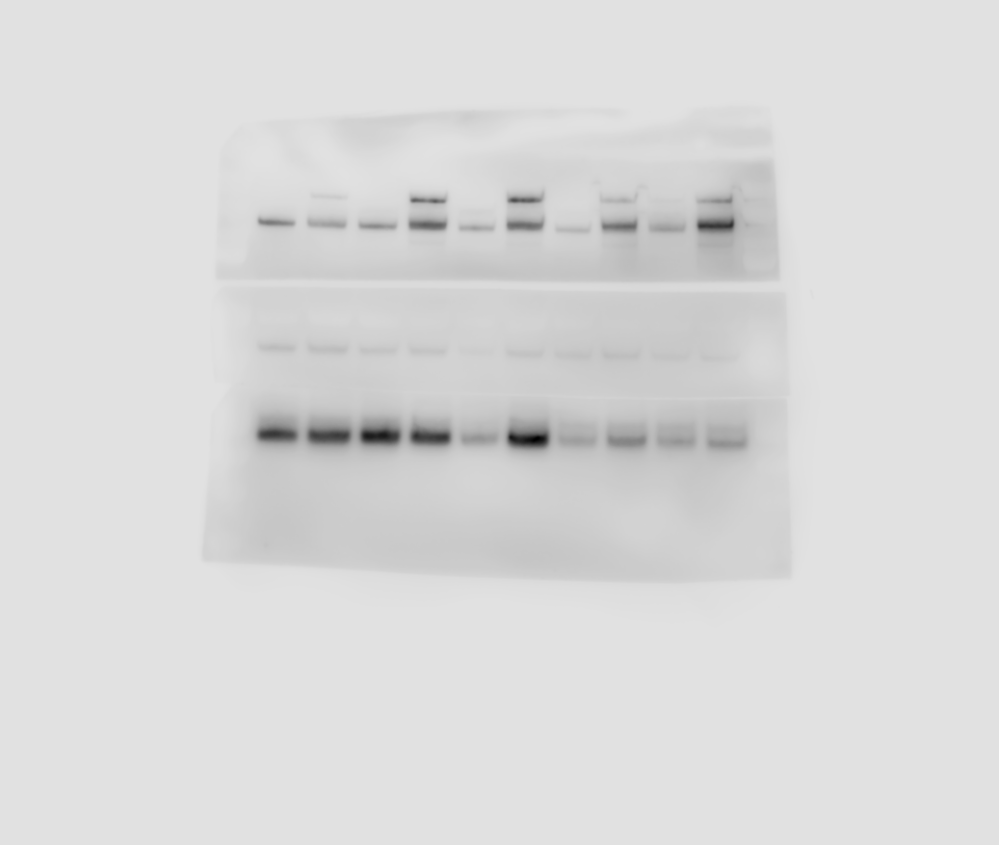

Supplement: Figure 4—figure supplement 1—source data 1. [file elife-101173-fig4-figsupp1-data1.zip › Figure 4-figure supplement 1-source data 1/090723_WB_DLK_cjun_Stmn4_membraneM_10min_2.tif]

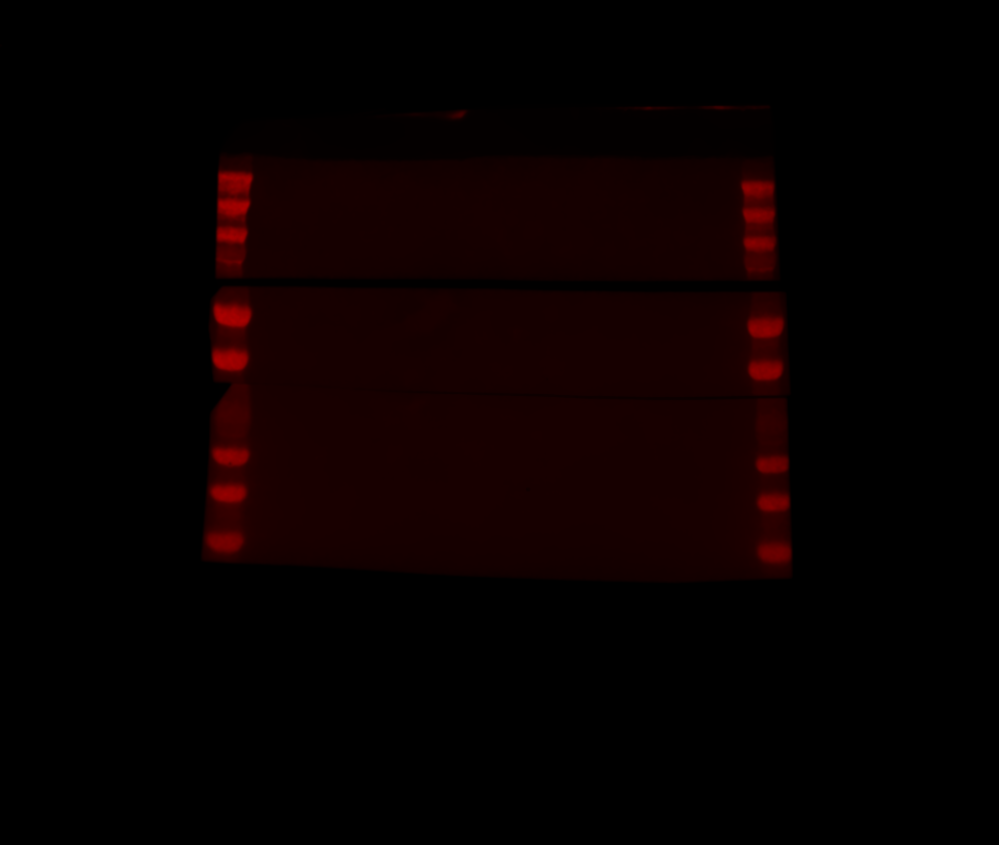

Supplement: Figure 4—figure supplement 1—source data 1. [file elife-101173-fig4-figsupp1-data1.zip › Figure 4-figure supplement 1-source data 1/090723_WB_DLK_cjun_Stmn4_membraneM_30sec_ladder.png]

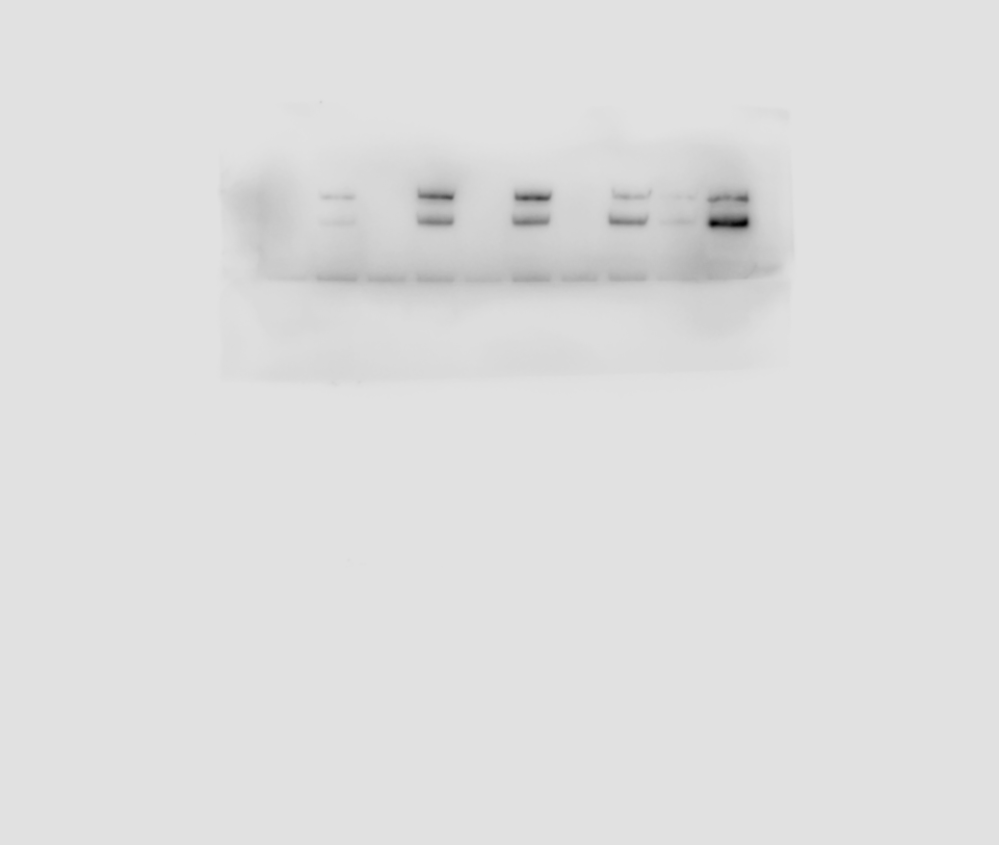

Supplement: Figure 4—figure supplement 1—source data 1. [file elife-101173-fig4-figsupp1-data1.zip › Figure 4-figure supplement 1-source data 1/090723_WB_Flag_pcjun_none_membraneN_2min.tif]

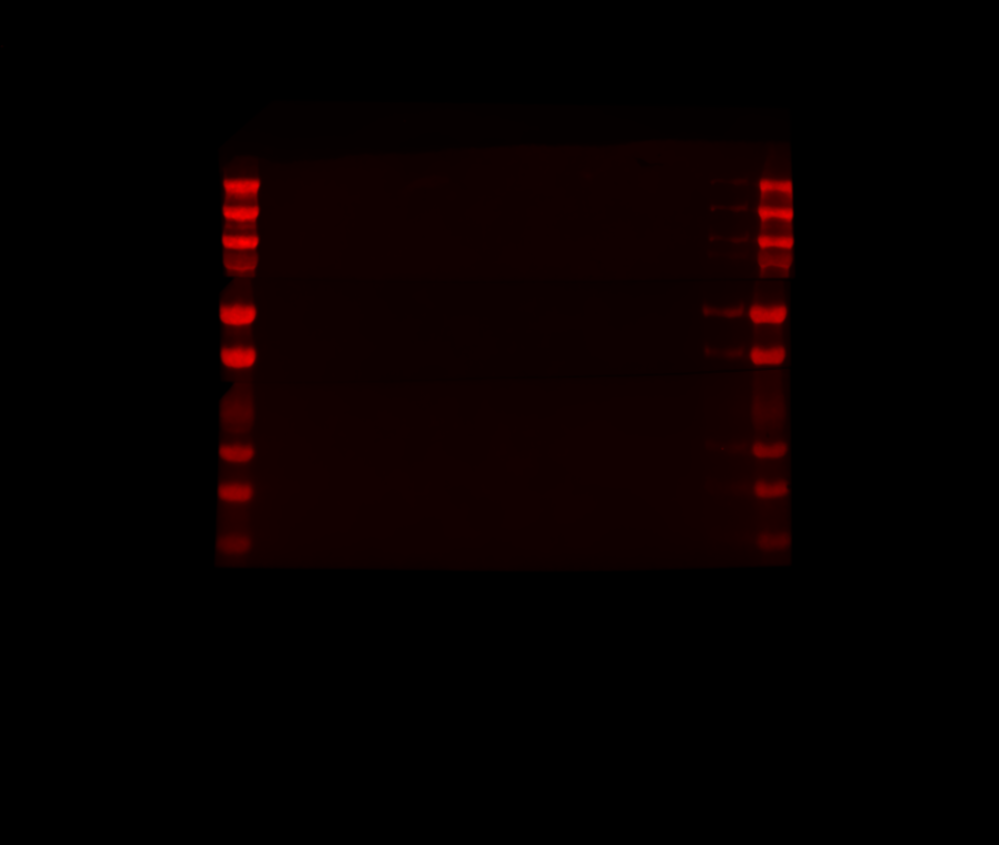

Supplement: Figure 4—figure supplement 1—source data 1. [file elife-101173-fig4-figsupp1-data1.zip › Figure 4-figure supplement 1-source data 1/090723_WB_Flag_pcjun_none_membraneN_30sec_ladder.tif]

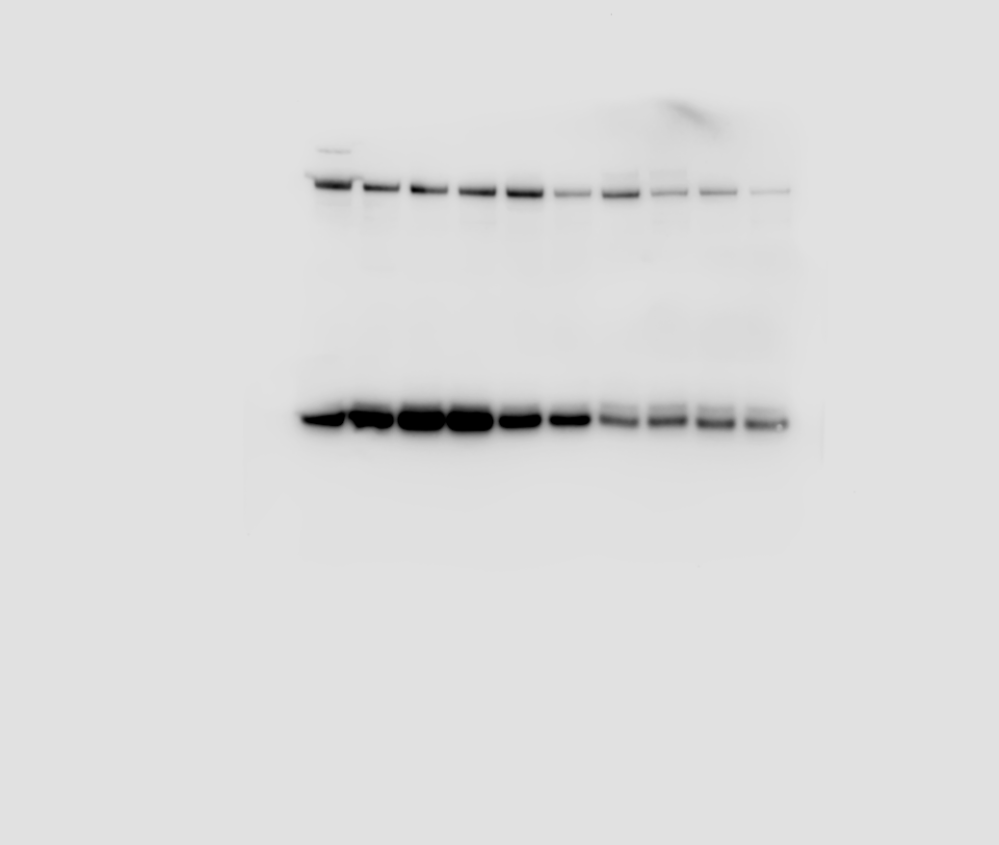

Supplement: Figure 4—figure supplement 1—source data 1. [file elife-101173-fig4-figsupp1-data1.zip › Figure 4-figure supplement 1-source data 1/091223_WB_DLK_pcjun_stmn4_membraneQ_10min.tif]

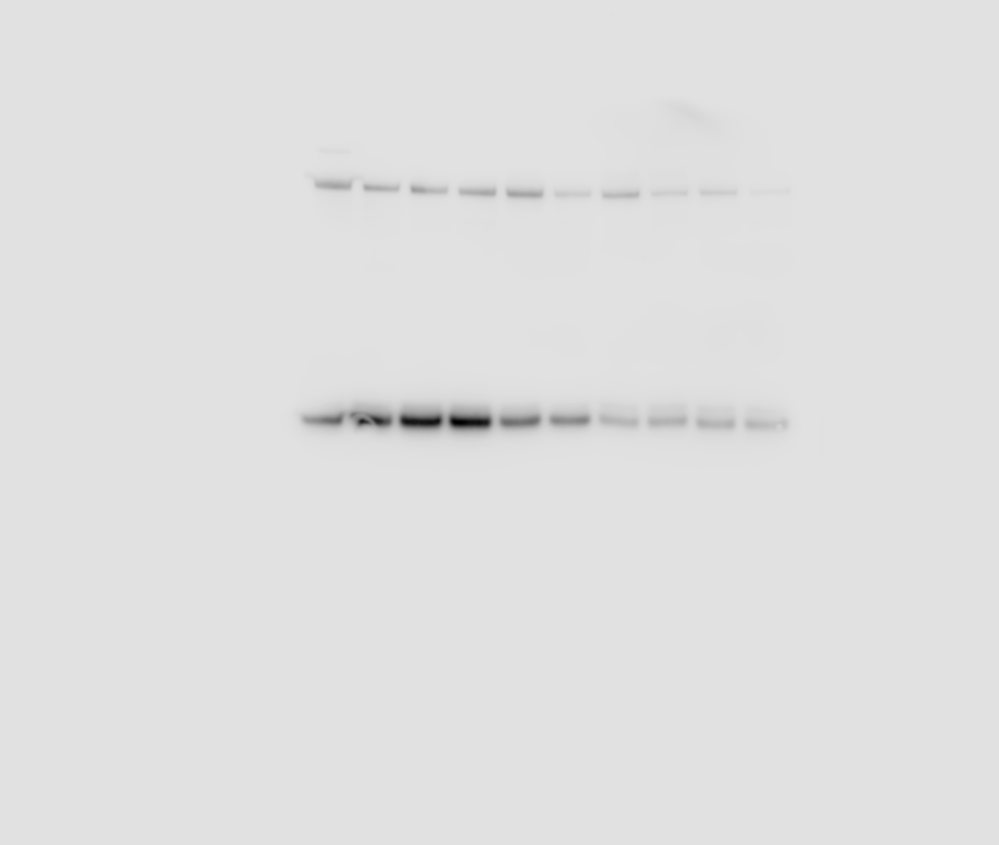

Supplement: Figure 4—figure supplement 1—source data 1. [file elife-101173-fig4-figsupp1-data1.zip › Figure 4-figure supplement 1-source data 1/091223_WB_DLK_pcjun_stmn4_membraneQ_10min2.tif]

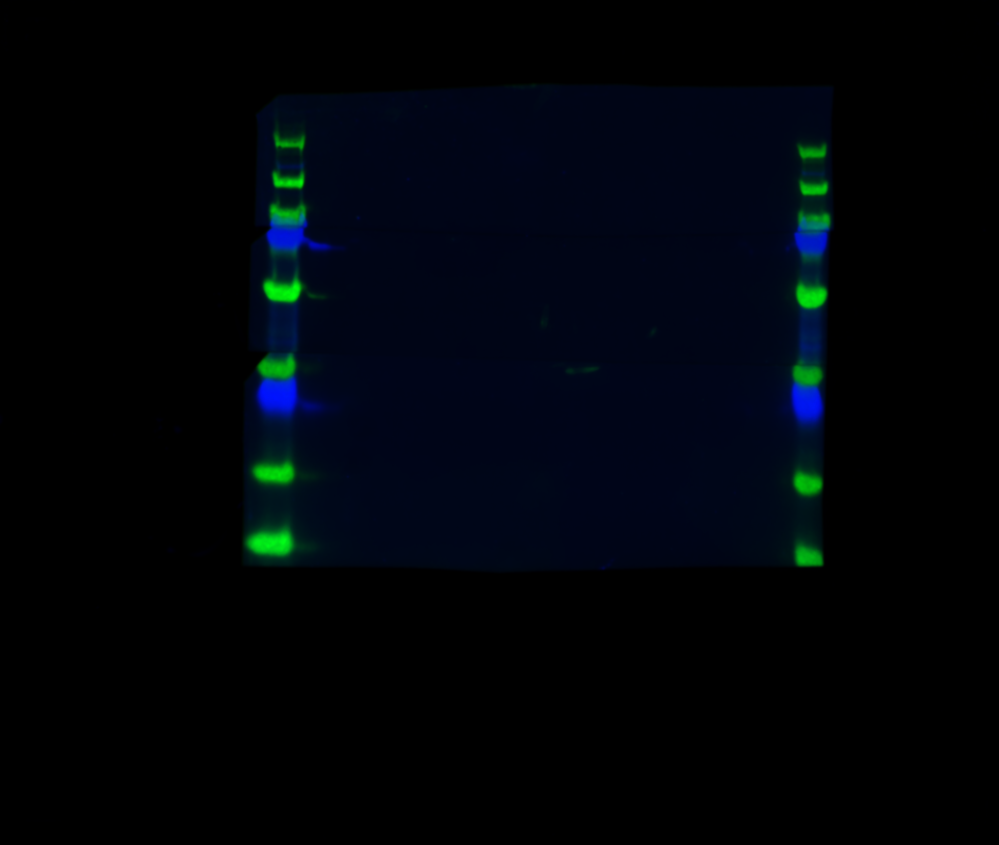

Supplement: Figure 4—figure supplement 1—source data 1. [file elife-101173-fig4-figsupp1-data1.zip › Figure 4-figure supplement 1-source data 1/091223_WB_DLK_pcjun_stmn4_membraneQ_ladder.tif]

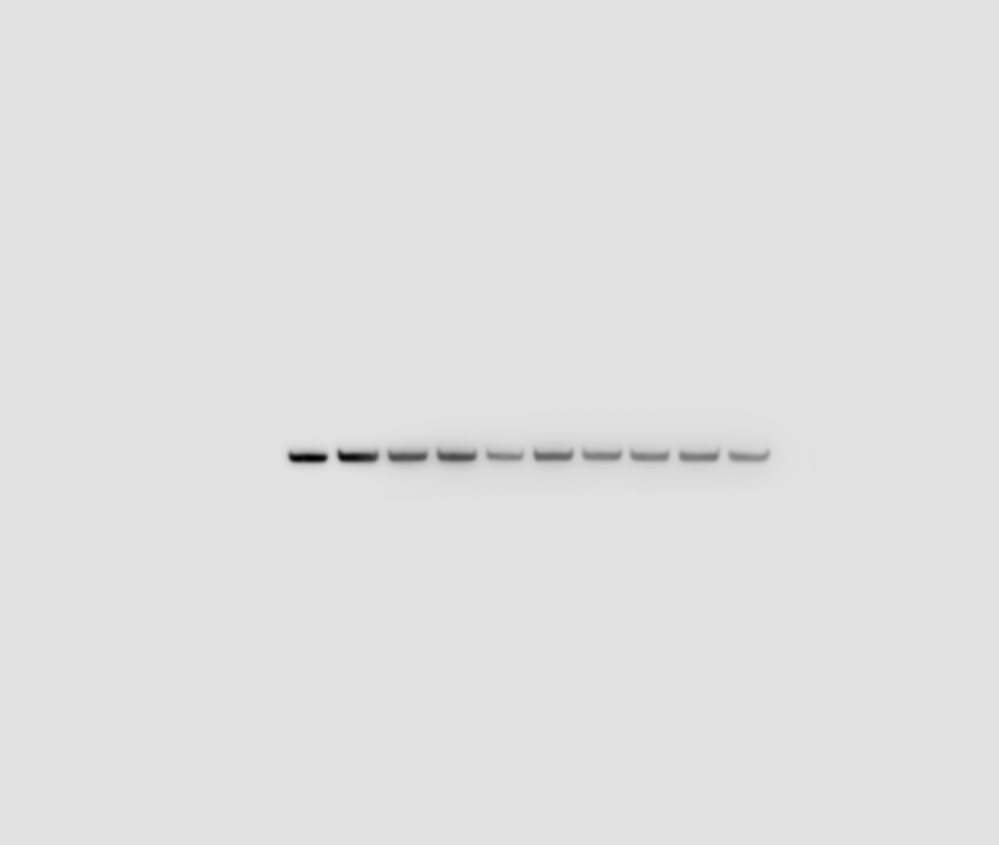

Supplement: Figure 4—figure supplement 1—source data 1. [file elife-101173-fig4-figsupp1-data1.zip › Figure 4-figure supplement 1-source data 1/091423_WB_actin_membraneN_2min.png]

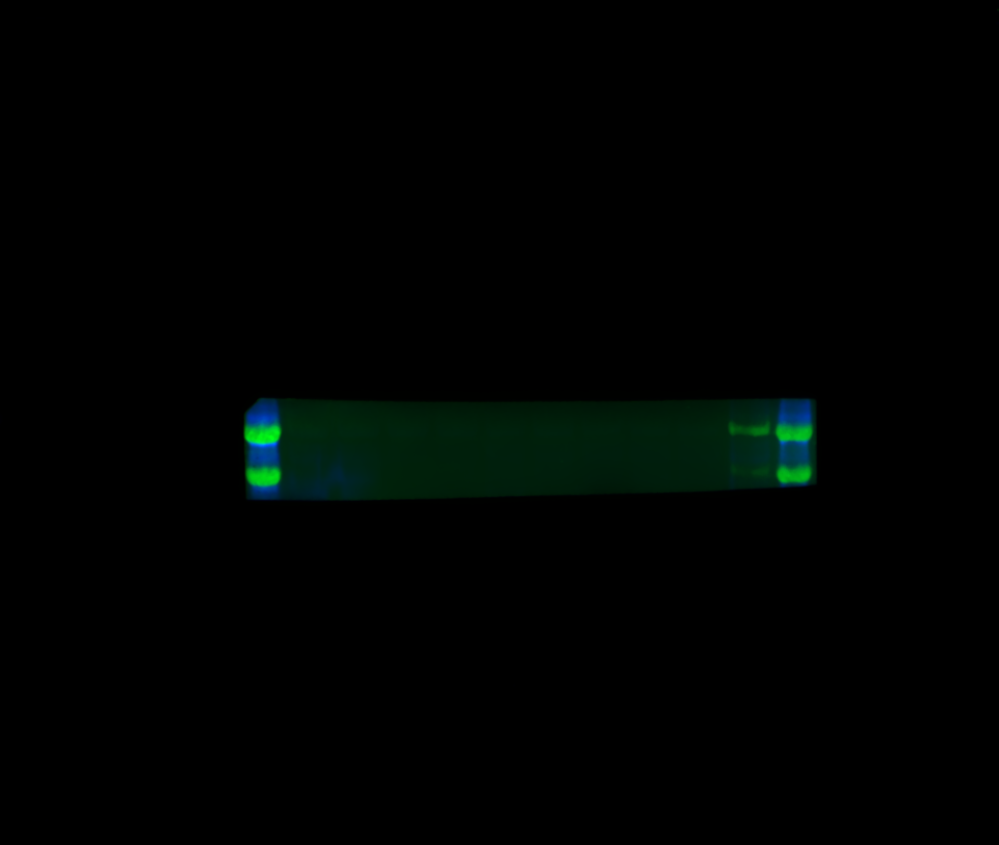

Supplement: Figure 4—figure supplement 1—source data 1. [file elife-101173-fig4-figsupp1-data1.zip › Figure 4-figure supplement 1-source data 1/091423_WB_actin_membraneN_ladder.tif]

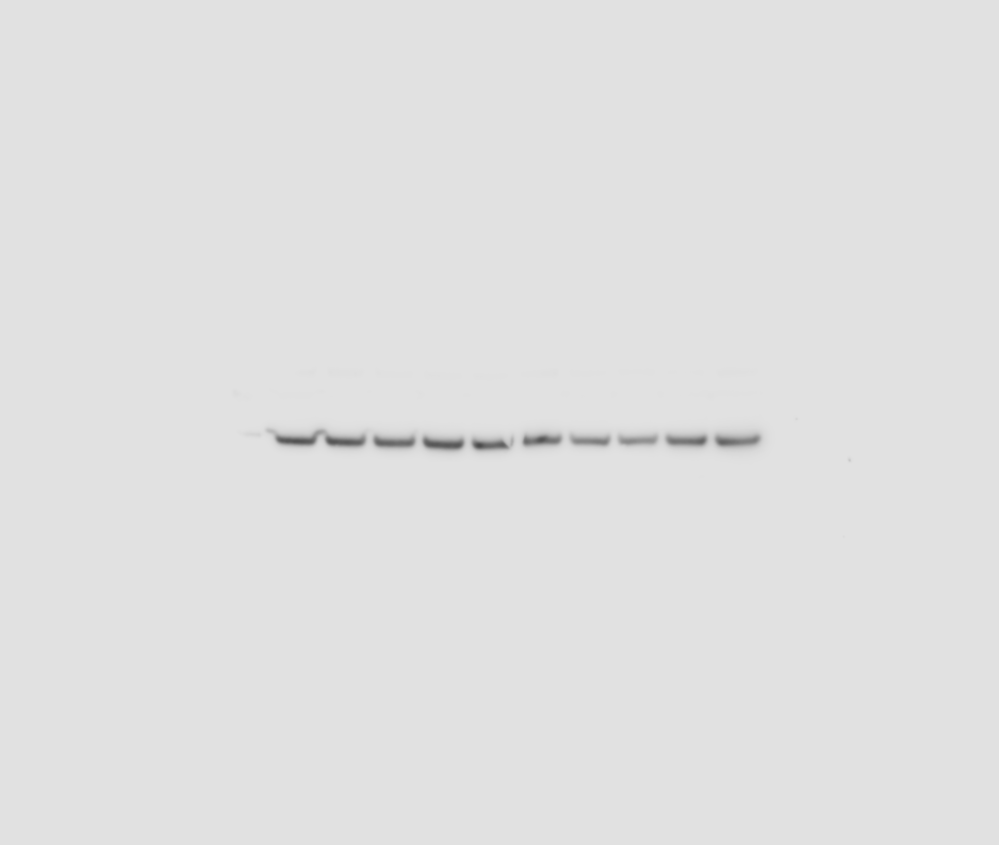

Supplement: Figure 4—figure supplement 1—source data 1. [file elife-101173-fig4-figsupp1-data1.zip › Figure 4-figure supplement 1-source data 1/092023_WB_actin_membraneQ_2min.tif]

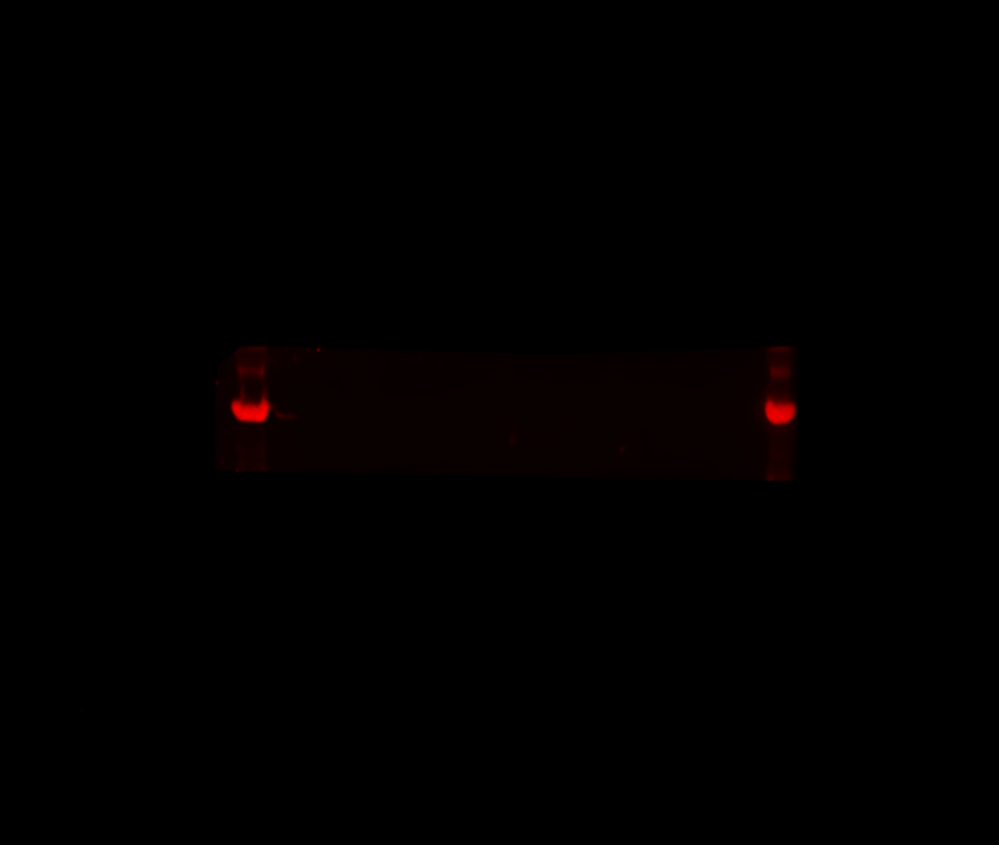

Supplement: Figure 4—figure supplement 1—source data 1. [file elife-101173-fig4-figsupp1-data1.zip › Figure 4-figure supplement 1-source data 1/092023_WB_actin_membraneQ_ladder.tif]

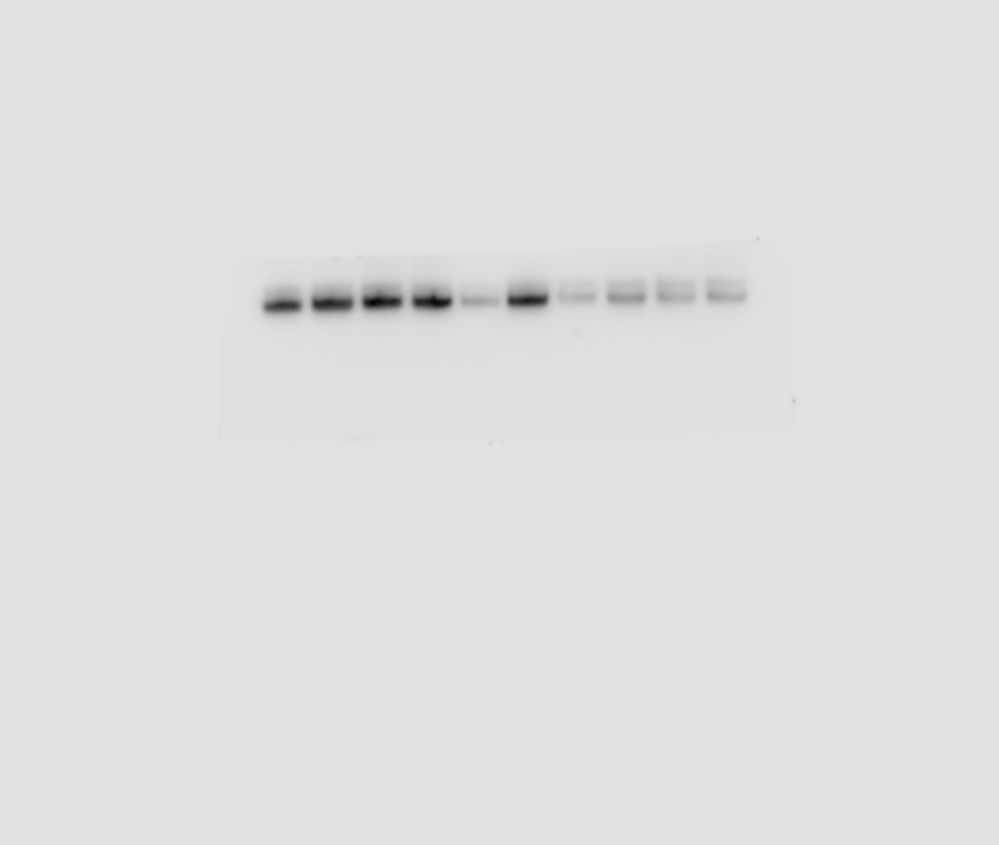

Supplement: Figure 4—figure supplement 1—source data 1. [file elife-101173-fig4-figsupp1-data1.zip › Figure 4-figure supplement 1-source data 1/102523_membrane N_stmn4_2min.tif]

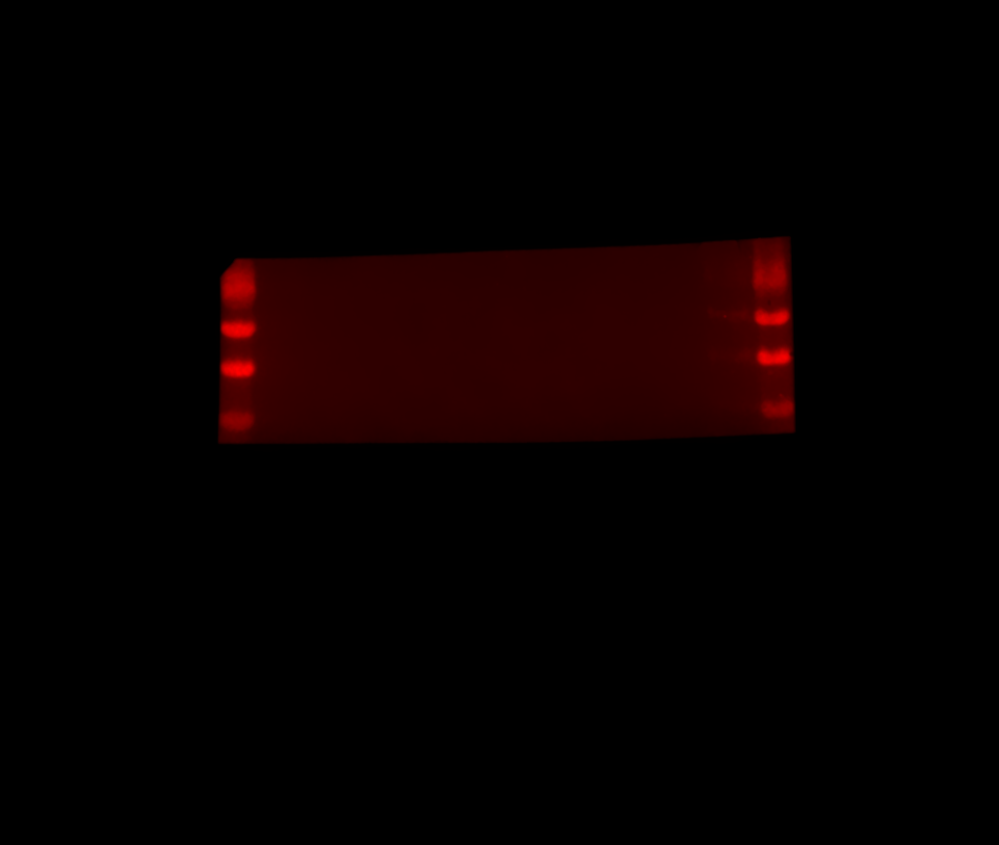

Supplement: Figure 4—figure supplement 1—source data 1. [file elife-101173-fig4-figsupp1-data1.zip › Figure 4-figure supplement 1-source data 1/102523_membrane N_stmn4_ladder.tif]

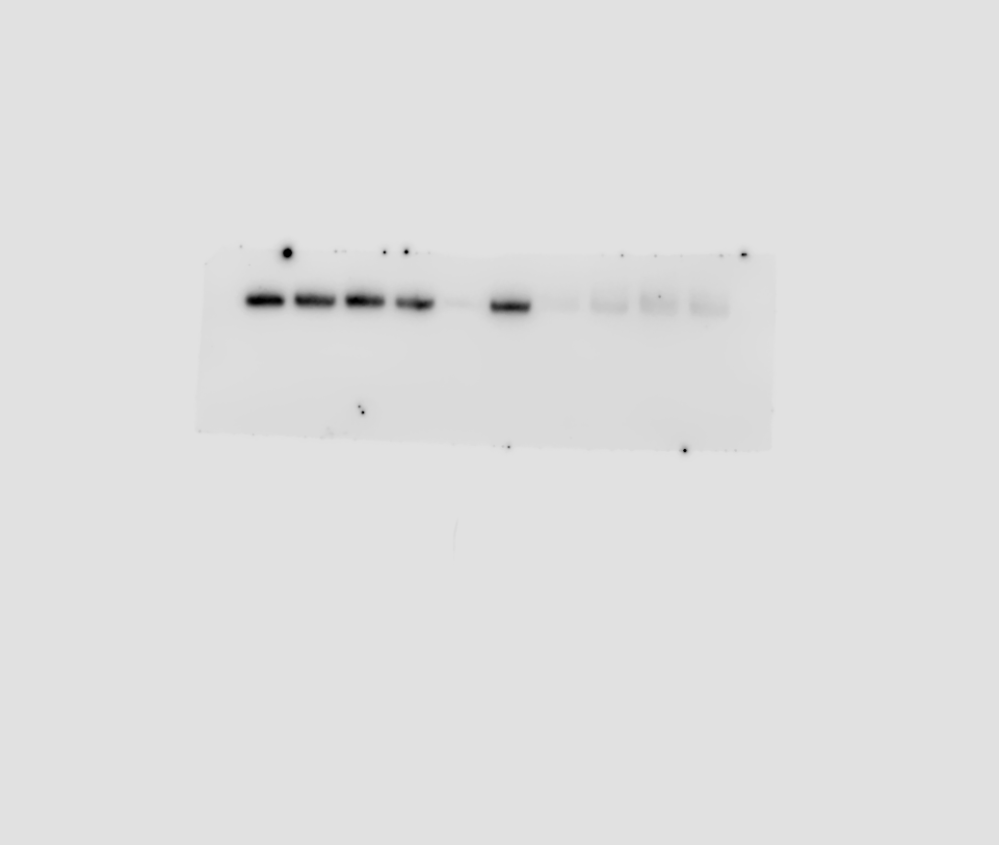

Supplement: Figure 4—figure supplement 1—source data 1. [file elife-101173-fig4-figsupp1-data1.zip › Figure 4-figure supplement 1-source data 1/102623_membrane N_stmn2_10min.tif]

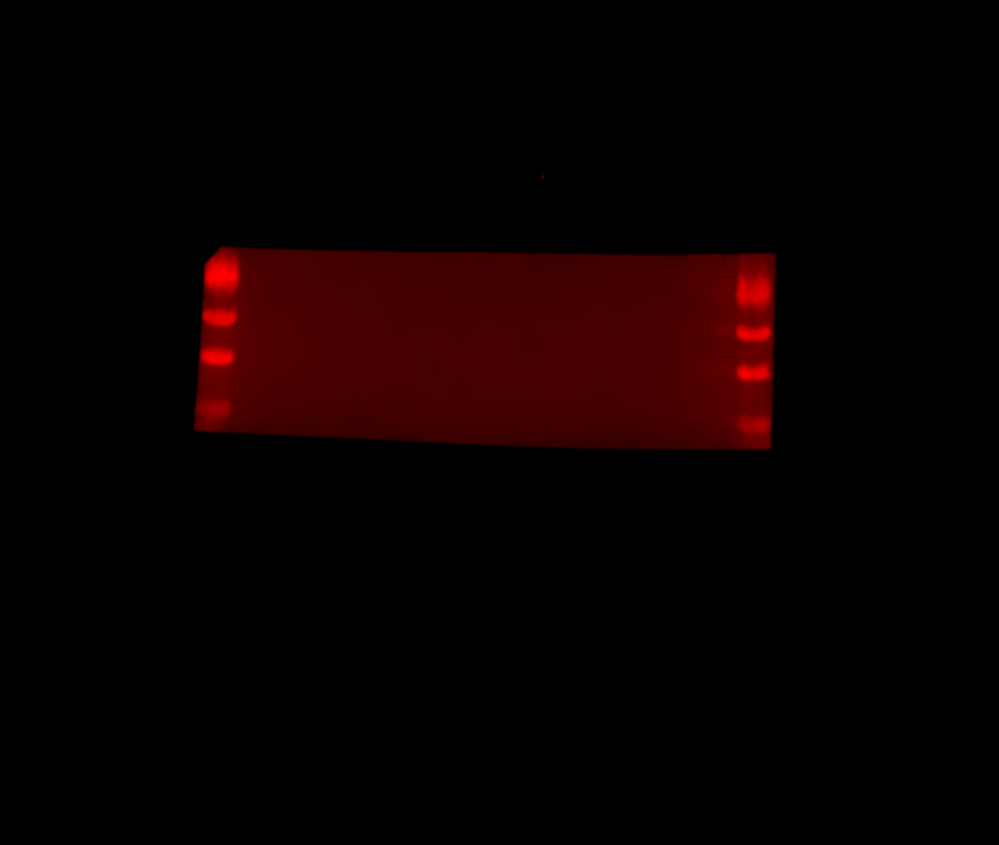

Supplement: Figure 4—figure supplement 1—source data 1. [file elife-101173-fig4-figsupp1-data1.zip › Figure 4-figure supplement 1-source data 1/102623_membrane N_stmn2_ladder.png]

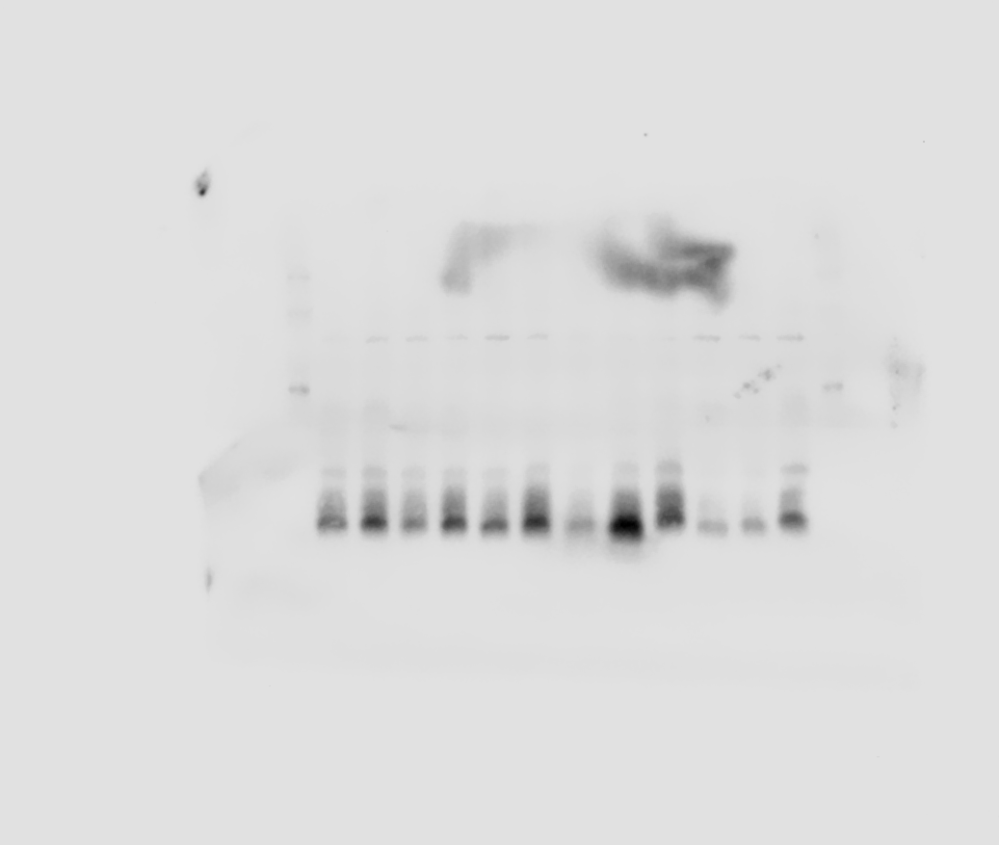

Supplement: Figure 6—figure supplement 2—source data 1. [file elife-101173-fig6-figsupp2-data1.zip › Figure 6-figure supplement 2-source data 1/010424_membraneU_SH2D3C-stmn4_10min1.tif]

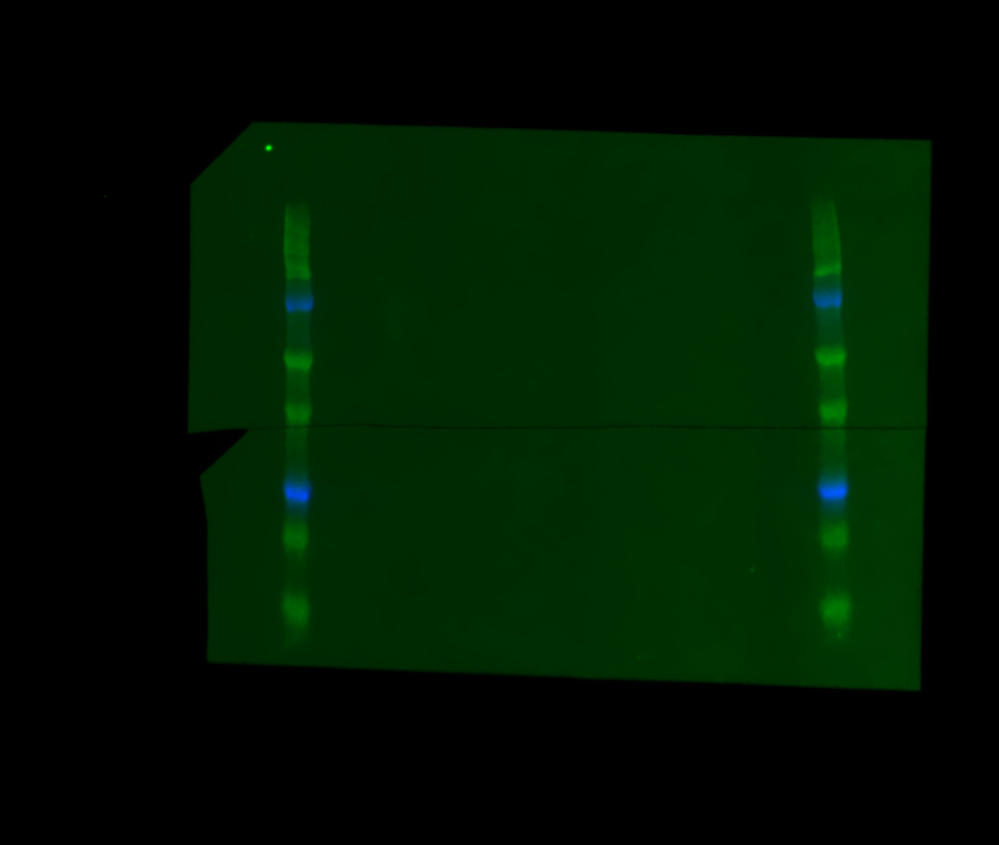

Supplement: Figure 6—figure supplement 2—source data 1. [file elife-101173-fig6-figsupp2-data1.zip › Figure 6-figure supplement 2-source data 1/010424_membraneU_SH2D3C-stmn4_ladder.tif]

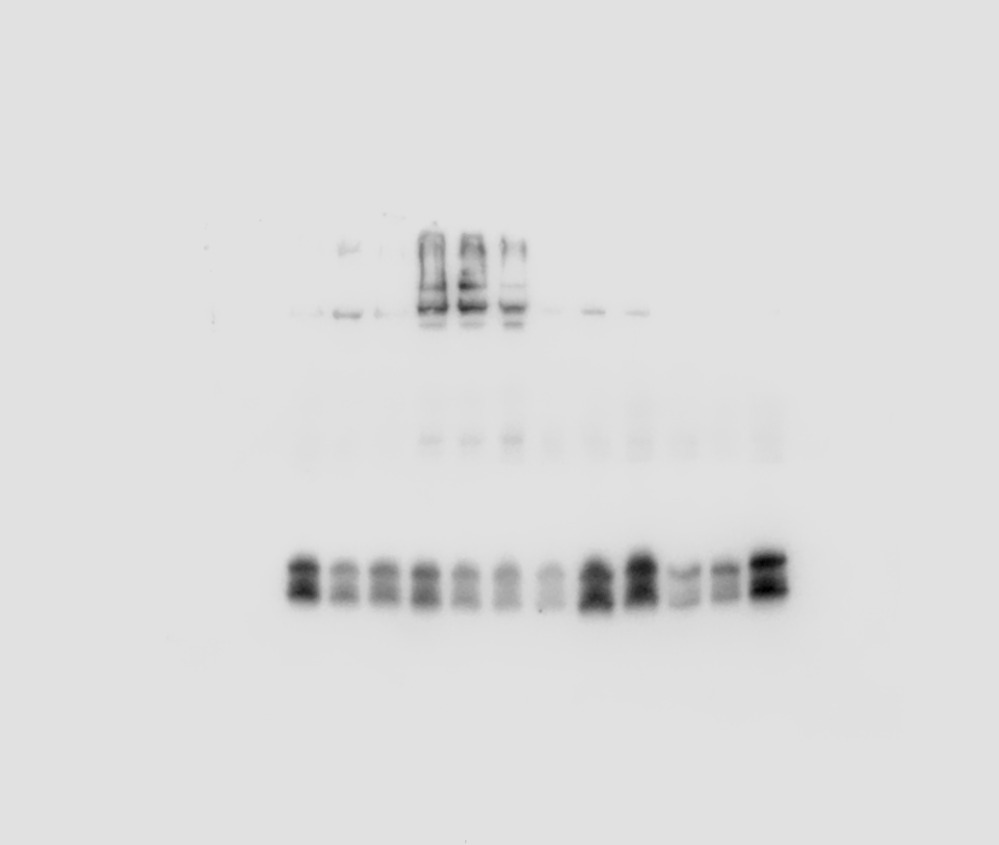

Supplement: Figure 6—figure supplement 2—source data 1. [file elife-101173-fig6-figsupp2-data1.zip › Figure 6-figure supplement 2-source data 1/010424_membraneV_DLK-Stmn2_2min.tif]

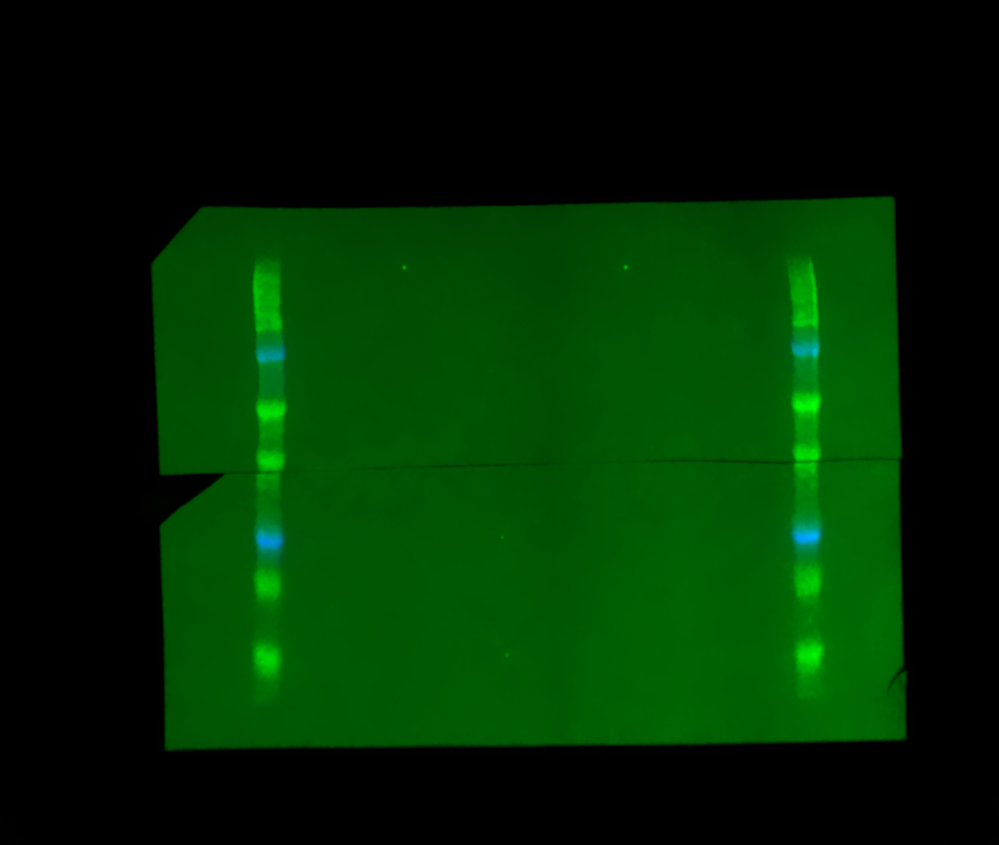

Supplement: Figure 6—figure supplement 2—source data 1. [file elife-101173-fig6-figsupp2-data1.zip › Figure 6-figure supplement 2-source data 1/010424_membraneV_DLK-Stmn2_ladder.tif]

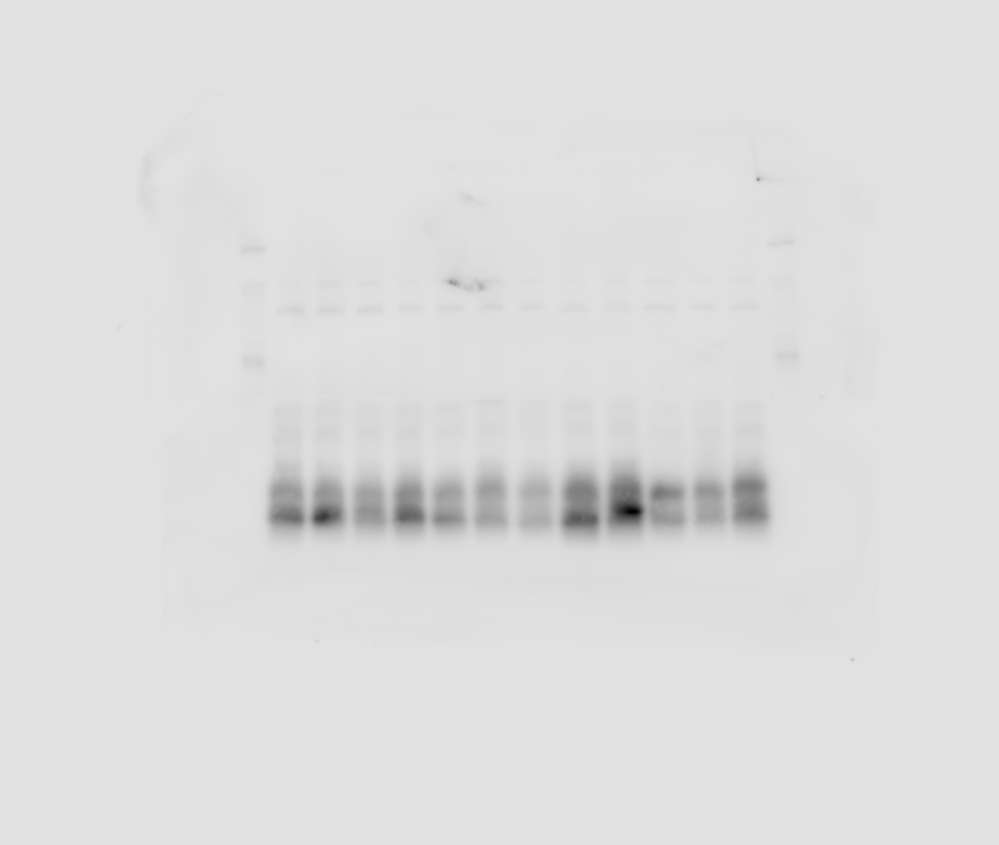

Supplement: Figure 6—figure supplement 2—source data 1. [file elife-101173-fig6-figsupp2-data1.zip › Figure 6-figure supplement 2-source data 1/010524_membraneU_Sh2d3c-Stmn2_10min2.tif]

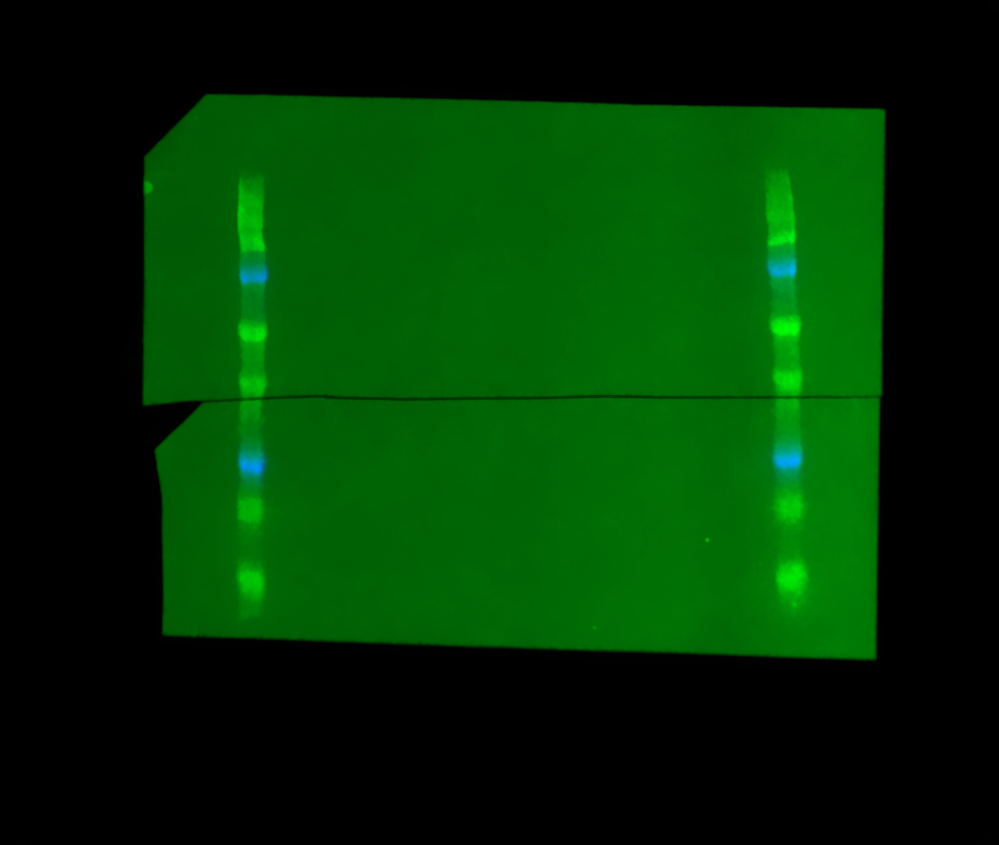

Supplement: Figure 6—figure supplement 2—source data 1. [file elife-101173-fig6-figsupp2-data1.zip › Figure 6-figure supplement 2-source data 1/010524_membraneU_Sh2d3c-Stmn2_ladder.tif]

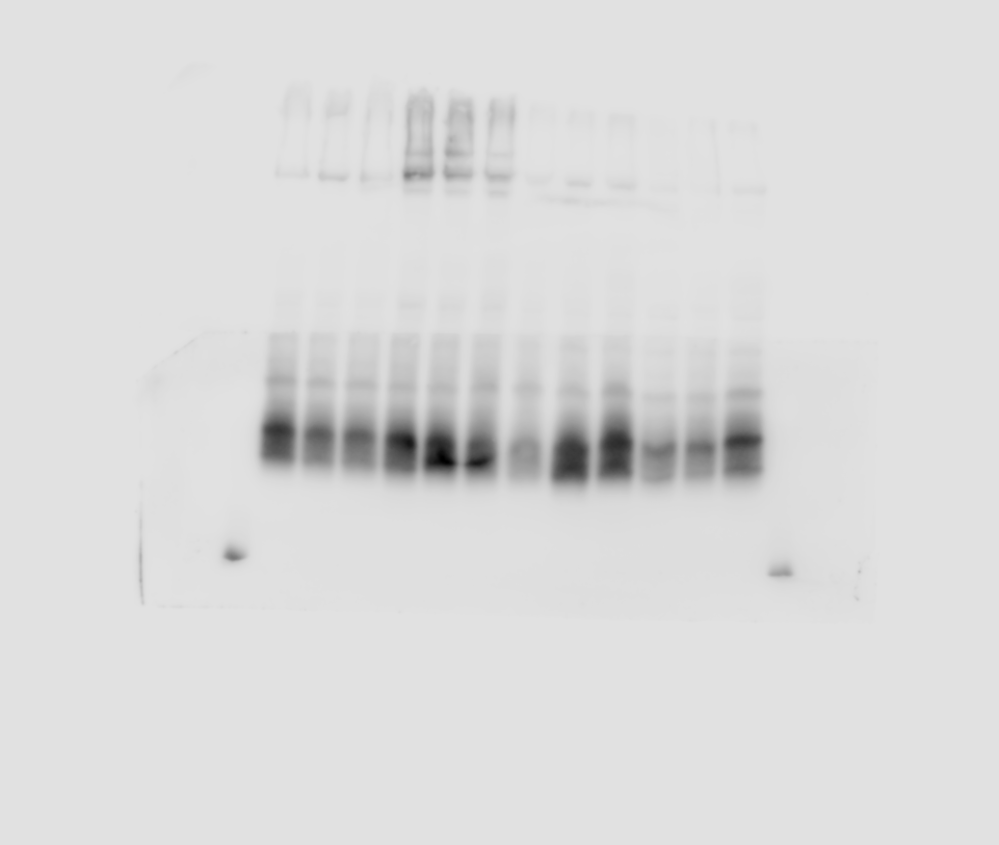

Supplement: Figure 6—figure supplement 2—source data 1. [file elife-101173-fig6-figsupp2-data1.zip › Figure 6-figure supplement 2-source data 1/010524_membraneV_DLK-Stmn4_10min.tif]

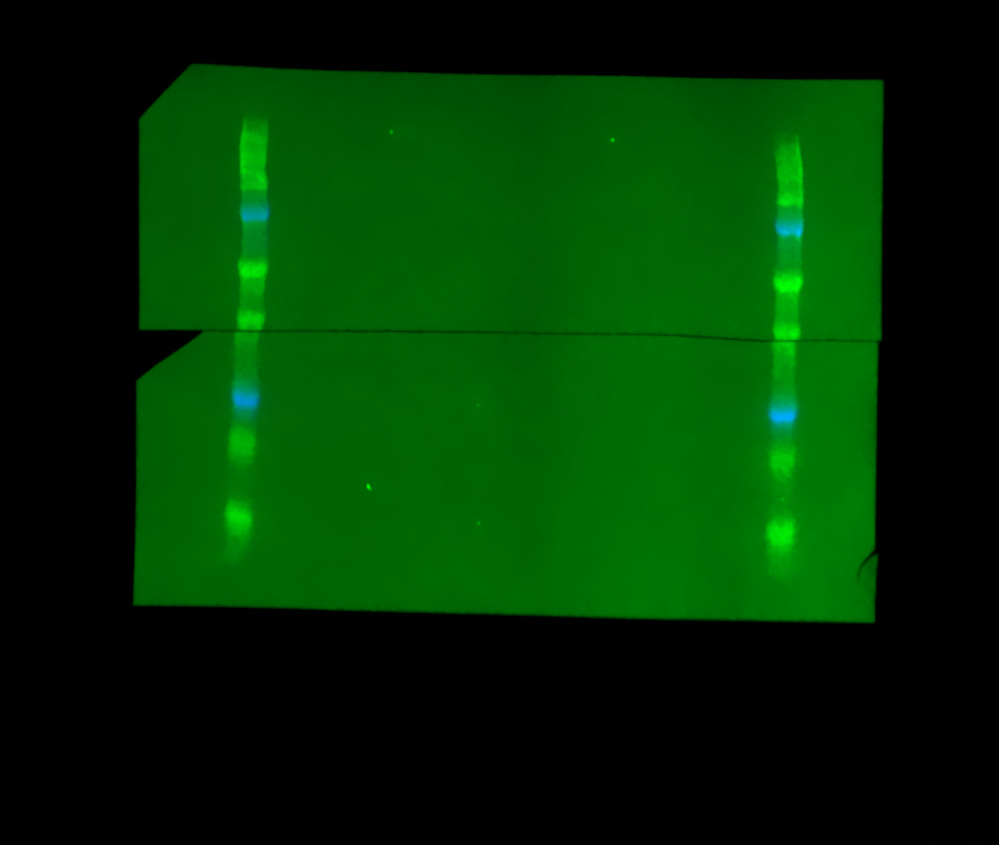

Supplement: Figure 6—figure supplement 2—source data 1. [file elife-101173-fig6-figsupp2-data1.zip › Figure 6-figure supplement 2-source data 1/010524_membraneV_DLK-Stmn4_ladder.tif]

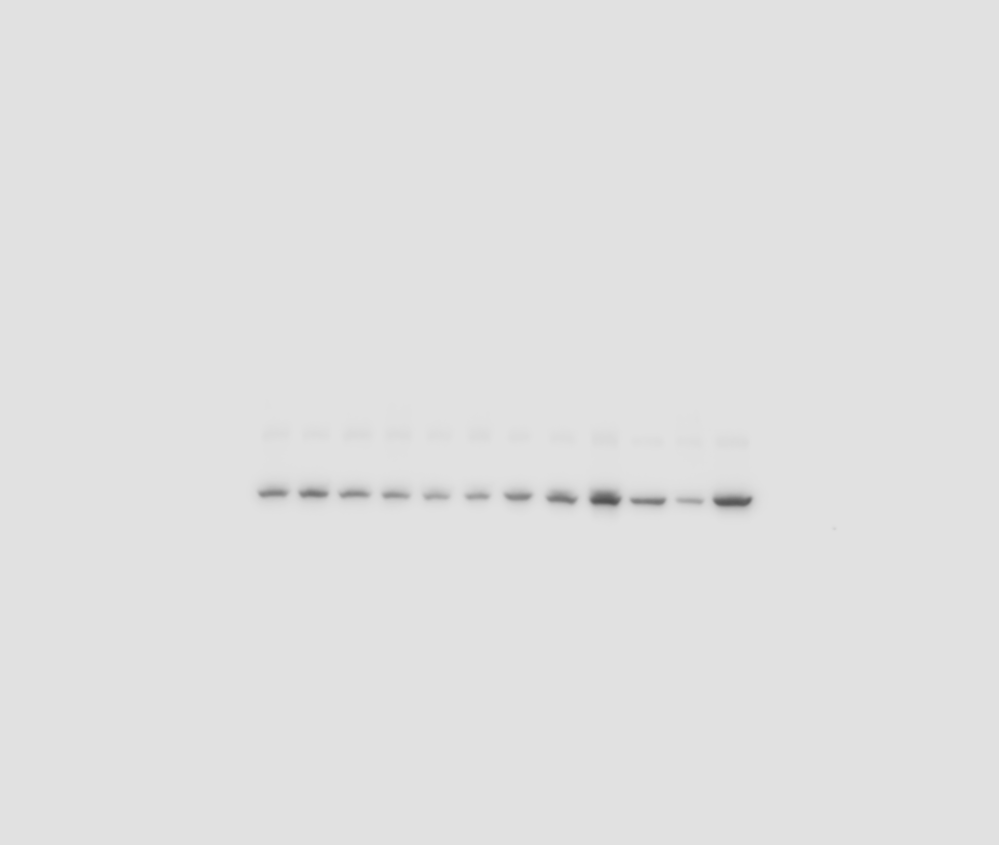

Supplement: Figure 6—figure supplement 2—source data 1. [file elife-101173-fig6-figsupp2-data1.zip › Figure 6-figure supplement 2-source data 1/010824_membraneV_actin_2min.tif]

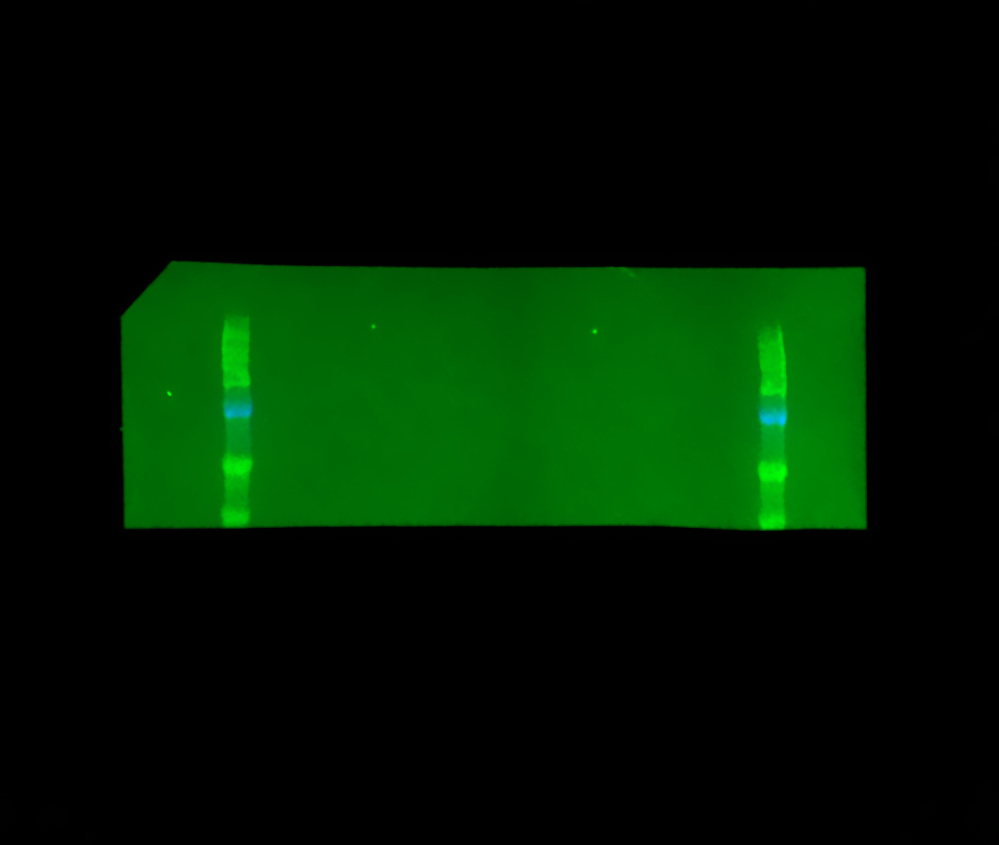

Supplement: Figure 6—figure supplement 2—source data 1. [file elife-101173-fig6-figsupp2-data1.zip › Figure 6-figure supplement 2-source data 1/010824_membraneV_actin_ladder.png]
