# Supplementary material for: Translatome analysis reveals cellular network in DLK-dependent hippocampal glutamatergic neuron degeneration
Source: eLife. 2025 Mar 11;13:RP101173. doi: 10.7554/eLife.101173 (PMC11896613; doi:10.7554/eLife.101173)
Supplement: Figure 1—source data 2. — Original membranes corresponding to Panel A. Molecular weights shown using PageRuler Plus Prestained Protein Ladder. See Figure 1—figure supplement 1—source data 2 for more details. Each lane represents a separate mouse. Lanes 1–3 show control samples, lanes 4–6 show DLK(cKO). [file elife-101173-fig1-data2.zip › Figure 1-source data 2/Figure 1-source data 2.pptx]

## Slide 1
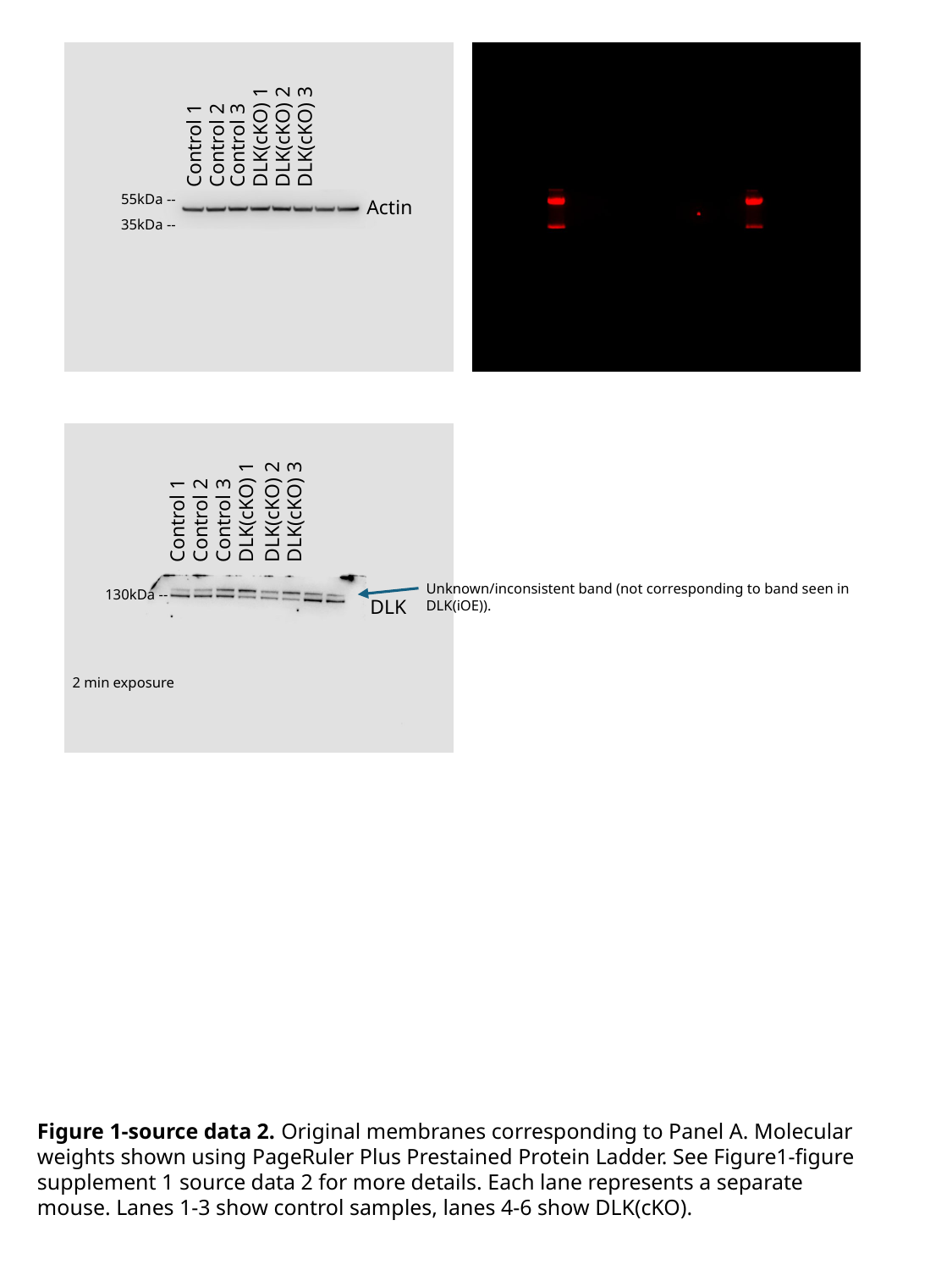

Control 1
Control 2
Control 3
DLK(cKO) 1
DLK(cKO) 2
DLK(cKO) 3
55kDa --
Actin
35kDa --
Control 1
Control 2
Control 3
DLK(cKO) 1
DLK(cKO) 2
DLK(cKO) 3
Unknown/inconsistent band (not corresponding to band seen in DLK(iOE)).
130kDa --
DLK
2 min exposure
Figure 1-source data 2. Original membranes corresponding to Panel A. Molecular weights shown using PageRuler Plus Prestained Protein Ladder. See Figure1-figure supplement 1 source data 2 for more details. Each lane represents a separate mouse. Lanes 1-3 show control samples, lanes 4-6 show DLK(cKO).
