# Supplementary material for: Translatome analysis reveals cellular network in DLK-dependent hippocampal glutamatergic neuron degeneration
Source: eLife. 2025 Mar 11;13:RP101173. doi: 10.7554/eLife.101173 (PMC11896613; doi:10.7554/eLife.101173)
Supplement: Figure 3—figure supplement 1—source data 2. — Original membranes corresponding to Panel A. Molecular weights shown using Precision Plus Protein Dual Color Ladder. Rpl22-HA is 23 kDa protein. [file elife-101173-fig3-figsupp1-data2.zip › Figure 3-figure supplement 1-source data 2/Figure 3-figure supplement 1-source data 2.pptx]

## Slide 1
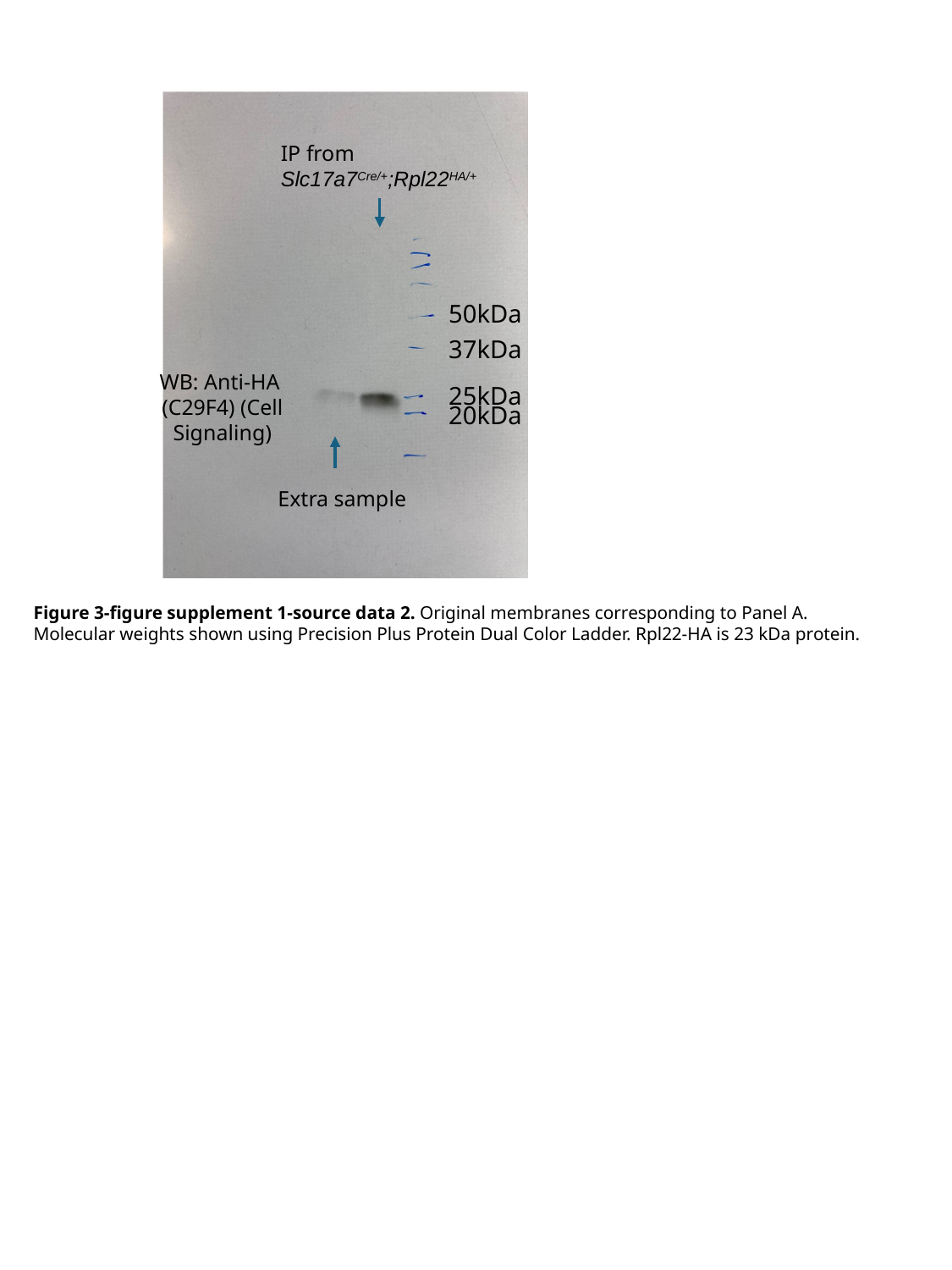

IP from
Slc17a7Cre/+;Rpl22HA/+
50kDa
37kDa
WB: Anti-HA
(C29F4) (Cell Signaling)
25kDa
20kDa
Extra sample
Figure 3-figure supplement 1-source data 2. Original membranes corresponding to Panel A. Molecular weights shown using Precision Plus Protein Dual Color Ladder. Rpl22-HA is 23 kDa protein.
