# Supplementary material for: Translatome analysis reveals cellular network in DLK-dependent hippocampal glutamatergic neuron degeneration
Source: eLife. 2025 Mar 11;13:RP101173. doi: 10.7554/eLife.101173 (PMC11896613; doi:10.7554/eLife.101173)
Supplement: Figure 4—figure supplement 1—source data 2. — Pg.1 Original membranes corresponding to Panel B. Molecular weights shown using PageRuler Plus Prestained Protein Ladder. Each lane represents a separate mouse. Lanes 1,3,5,7,9 show control samples, lanes 2,4,6,8,10 show DLK(cKO) at P1, P8, P15, P60, and 1 year timepoints. Dotted lines indicate locations where membrane was cut and labeled with separate antibodies. Pg.2 Original membranes corresponding to Panel C. Molecular weights shown using Precision Plus Protein Dual Color Ladder. Each lane represents a separate mouse. Lanes 1,3,5,7,9 show control samples, lanes 2,4,6,8,10 show DLK(iOE) at P1, P8, P15, P60, and ~1 year timepoints. Dotted lines indicate locations where membrane was cut and labeled with separate antibodies. Samples were split, with half of each prepped sample loaded onto two membranes (membrane 1&2). Smaller molecular weight band matches expected size of DLK protein (and flag tagged DLK). Larger molecular weight band of DLK in Vglut1Cre/+;H11-DLKiOE/+ would match the predicted molecular weight of DLK-T2A-tdTomato if T2A-peptide induced ‘self- cleavage’ due to ribosomal skipping is ineffective. Pg.3 Original membranes corresponding to Panel C. Molecular weights shown using Precision Plus Protein Dual Color Ladder. Each lane represents a separate mouse. Lanes 1,3,5,7,9 show control samples, lanes 2,4,6,8,10 show DLK(iOE) at P1, P8, P15, P60, and ~1 year timepoints. [file elife-101173-fig4-figsupp1-data2.zip › Figure 4-figure supplement 1-source data 2/Figure 4-figure supplement 1-source data 2.pptx]

## Slide 1
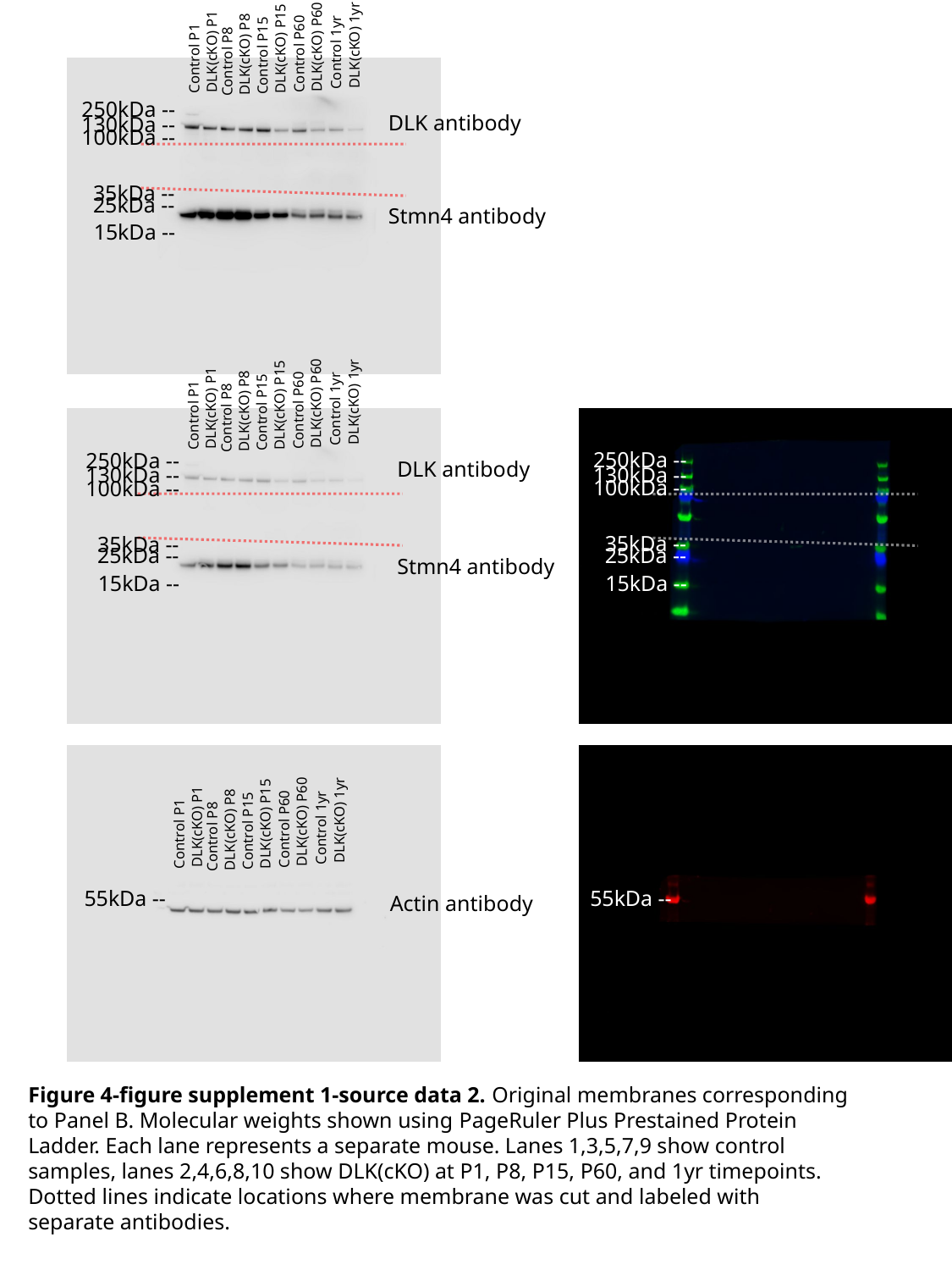

DLK(cKO) 1yr
Control 1yr
DLK(cKO) P60
DLK(cKO) P1
Control P60
Control P1
DLK(cKO) P15
Control P15
DLK(cKO) P8
Control P8
250kDa --
DLK antibody
130kDa --
100kDa --
35kDa --
25kDa --
Stmn4 antibody
15kDa --
DLK(cKO) 1yr
Control 1yr
DLK(cKO) P60
DLK(cKO) P1
Control P60
Control P1
DLK(cKO) P15
Control P15
DLK(cKO) P8
Control P8
250kDa --
250kDa --
DLK antibody
130kDa --
130kDa --
100kDa --
100kDa --
35kDa --
35kDa --
25kDa --
25kDa --
Stmn4 antibody
15kDa --
15kDa --
DLK(cKO) 1yr
Control 1yr
DLK(cKO) P60
DLK(cKO) P1
Control P60
Control P1
DLK(cKO) P15
Control P15
DLK(cKO) P8
Control P8
55kDa --
55kDa --
Actin antibody
Figure 4-figure supplement 1-source data 2. Original membranes corresponding to Panel B. Molecular weights shown using PageRuler Plus Prestained Protein Ladder. Each lane represents a separate mouse. Lanes 1,3,5,7,9 show control samples, lanes 2,4,6,8,10 show DLK(cKO) at P1, P8, P15, P60, and 1yr timepoints. Dotted lines indicate locations where membrane was cut and labeled with separate antibodies.

## Slide 2
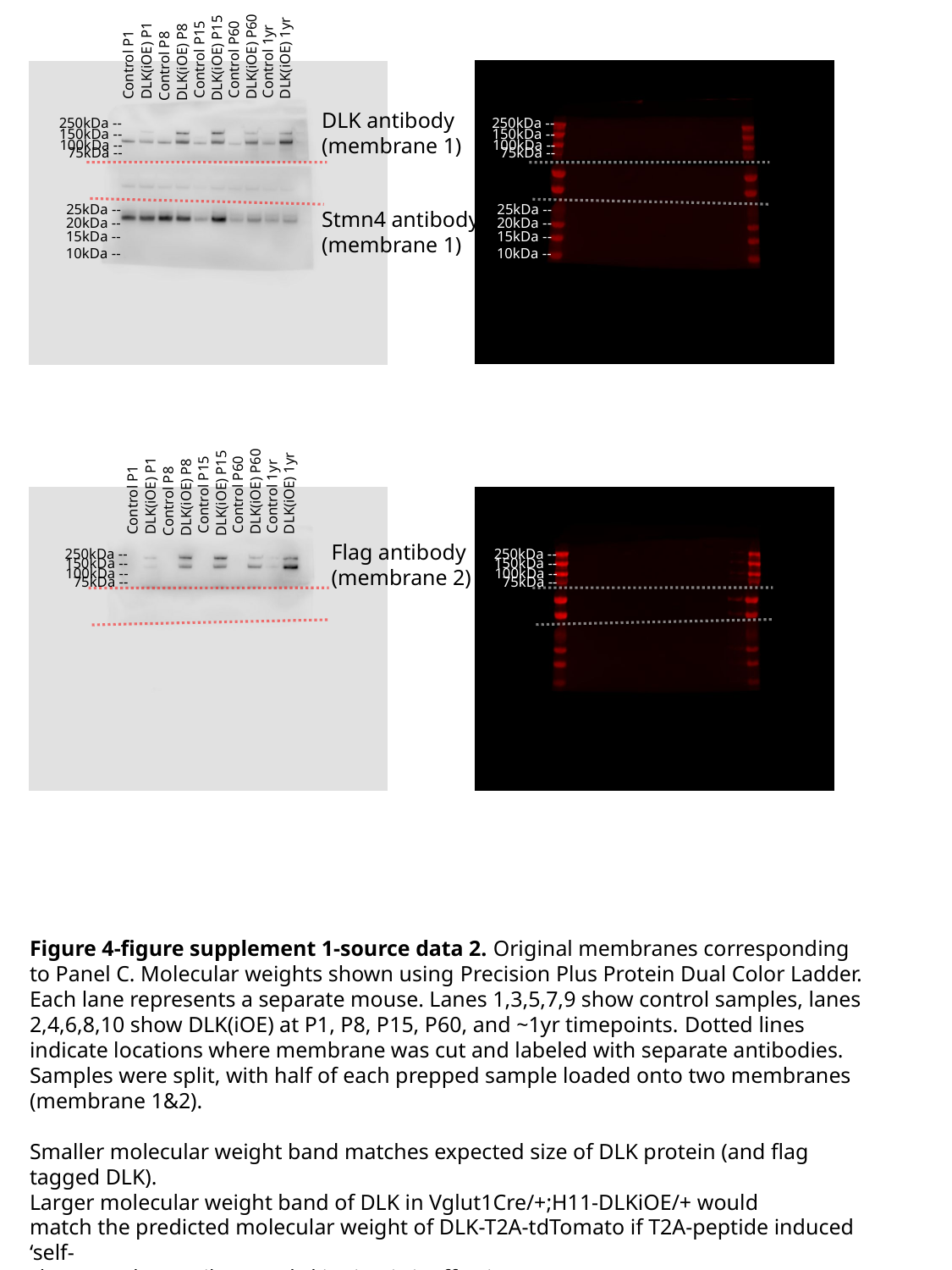

Control P15
Control P60
Control 1yr
DLK(iOE) 1yr
Control P1
DLK(iOE) P1
DLK(iOE) P60
Control P8
DLK(iOE) P8
DLK(iOE) P15
DLK antibody
(membrane 1)
250kDa --
250kDa --
150kDa --
150kDa --
100kDa --
100kDa --
75kDa --
75kDa --
25kDa --
25kDa --
Stmn4 antibody
(membrane 1)
20kDa --
20kDa --
15kDa --
15kDa --
10kDa --
10kDa --
Control P15
Control P60
Control 1yr
DLK(iOE) 1yr
Control P1
DLK(iOE) P1
DLK(iOE) P60
Control P8
DLK(iOE) P8
DLK(iOE) P15
Flag antibody
(membrane 2)
250kDa --
250kDa --
150kDa --
150kDa --
100kDa --
100kDa --
75kDa --
75kDa --
Figure 4-figure supplement 1-source data 2. Original membranes corresponding to Panel C. Molecular weights shown using Precision Plus Protein Dual Color Ladder. Each lane represents a separate mouse. Lanes 1,3,5,7,9 show control samples, lanes 2,4,6,8,10 show DLK(iOE) at P1, P8, P15, P60, and ~1yr timepoints. Dotted lines indicate locations where membrane was cut and labeled with separate antibodies. Samples were split, with half of each prepped sample loaded onto two membranes (membrane 1&2).
Smaller molecular weight band matches expected size of DLK protein (and flag tagged DLK).
Larger molecular weight band of DLK in Vglut1Cre/+;H11-DLKiOE/+ would
match the predicted molecular weight of DLK-T2A-tdTomato if T2A-peptide induced ‘self-
cleavage’ due to ribosomal skipping is ineffective.

## Slide 3
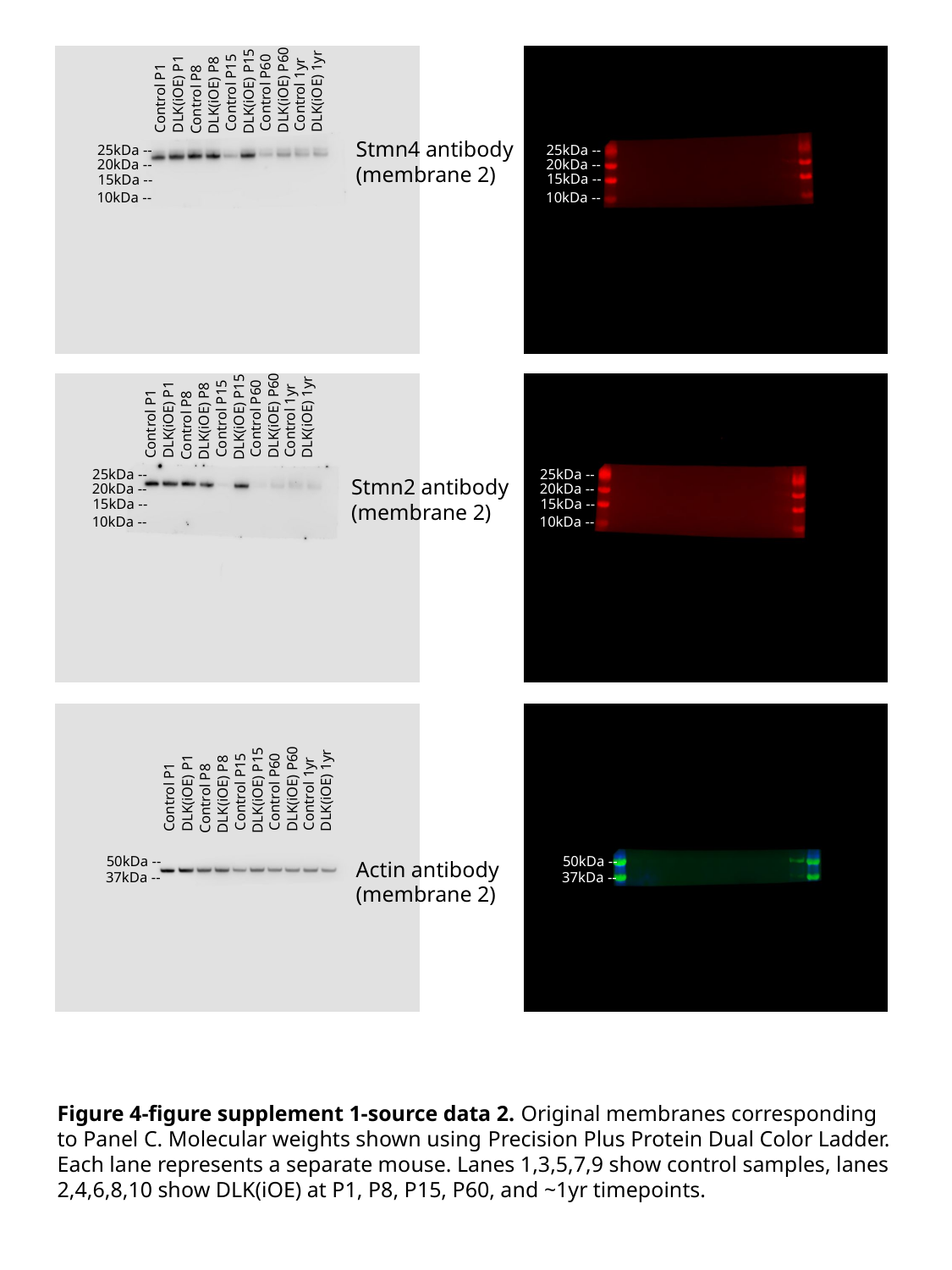

Control P15
Control P60
Control 1yr
DLK(iOE) 1yr
Control P1
DLK(iOE) P1
DLK(iOE) P60
Control P8
DLK(iOE) P8
DLK(iOE) P15
Stmn4 antibody
(membrane 2)
25kDa --
25kDa --
20kDa --
20kDa --
15kDa --
15kDa --
10kDa --
10kDa --
Control P15
Control P60
Control 1yr
DLK(iOE) 1yr
Control P1
DLK(iOE) P1
DLK(iOE) P60
Control P8
DLK(iOE) P8
DLK(iOE) P15
25kDa --
25kDa --
Stmn2 antibody
(membrane 2)
20kDa --
20kDa --
15kDa --
15kDa --
10kDa --
10kDa --
Control P15
Control P60
Control 1yr
DLK(iOE) 1yr
Control P1
DLK(iOE) P1
DLK(iOE) P60
Control P8
DLK(iOE) P8
DLK(iOE) P15
50kDa --
50kDa --
Actin antibody
(membrane 2)
37kDa --
37kDa --
Figure 4-figure supplement 1-source data 2. Original membranes corresponding to Panel C. Molecular weights shown using Precision Plus Protein Dual Color Ladder. Each lane represents a separate mouse. Lanes 1,3,5,7,9 show control samples, lanes 2,4,6,8,10 show DLK(iOE) at P1, P8, P15, P60, and ~1yr timepoints.
