# Supplementary material for: Translatome analysis reveals cellular network in DLK-dependent hippocampal glutamatergic neuron degeneration
Source: eLife. 2025 Mar 11;13:RP101173. doi: 10.7554/eLife.101173 (PMC11896613; doi:10.7554/eLife.101173)
Supplement: Figure 6—figure supplement 2—source data 2. — Pg. 1 Original membranes corresponding to Panel B. Molecular weights shown using Precision Plus Protein Dual Color Ladder. Each lane represents a separate mouse. Lanes 1–3 show control samples (from DLK(iOE) sibs), lanes 4–6 show DLK(iOE), lanes 7–9 show control samples (from DLK(cKO) sibs), lanes 10–12 show DLK(cKO). All from P15 timepoint. Dotted lines indicate locations where membrane was cut for labeling with separate antibodies. Samples were split, with half of each prepped sample loaded onto two membranes (membrane 1&2). Pg. 2 Original membranes corresponding to Panel B. Molecular weights shown using Precision Plus Protein Dual Color Ladder. Each lane represents a separate mouse. Lanes 1–3 show control samples (from DLK(iOE) sibs), lanes 4–6 show DLK(iOE), lanes 7–9 show control samples (from DLK(cKO) sibs), lanes 10–12 show DLK(cKO). All from P15 timepoint. Dotted lines indicate locations where membrane was cut for labeling with separate antibodies. Samples were split, with half of each prepped sample loaded onto two membranes (membrane 1&2). [file elife-101173-fig6-figsupp2-data2.zip › Figure 6-figure supplement 2-source data 2/Figure 6-figure supplement 2-source data 2.pptx]

## Slide 1
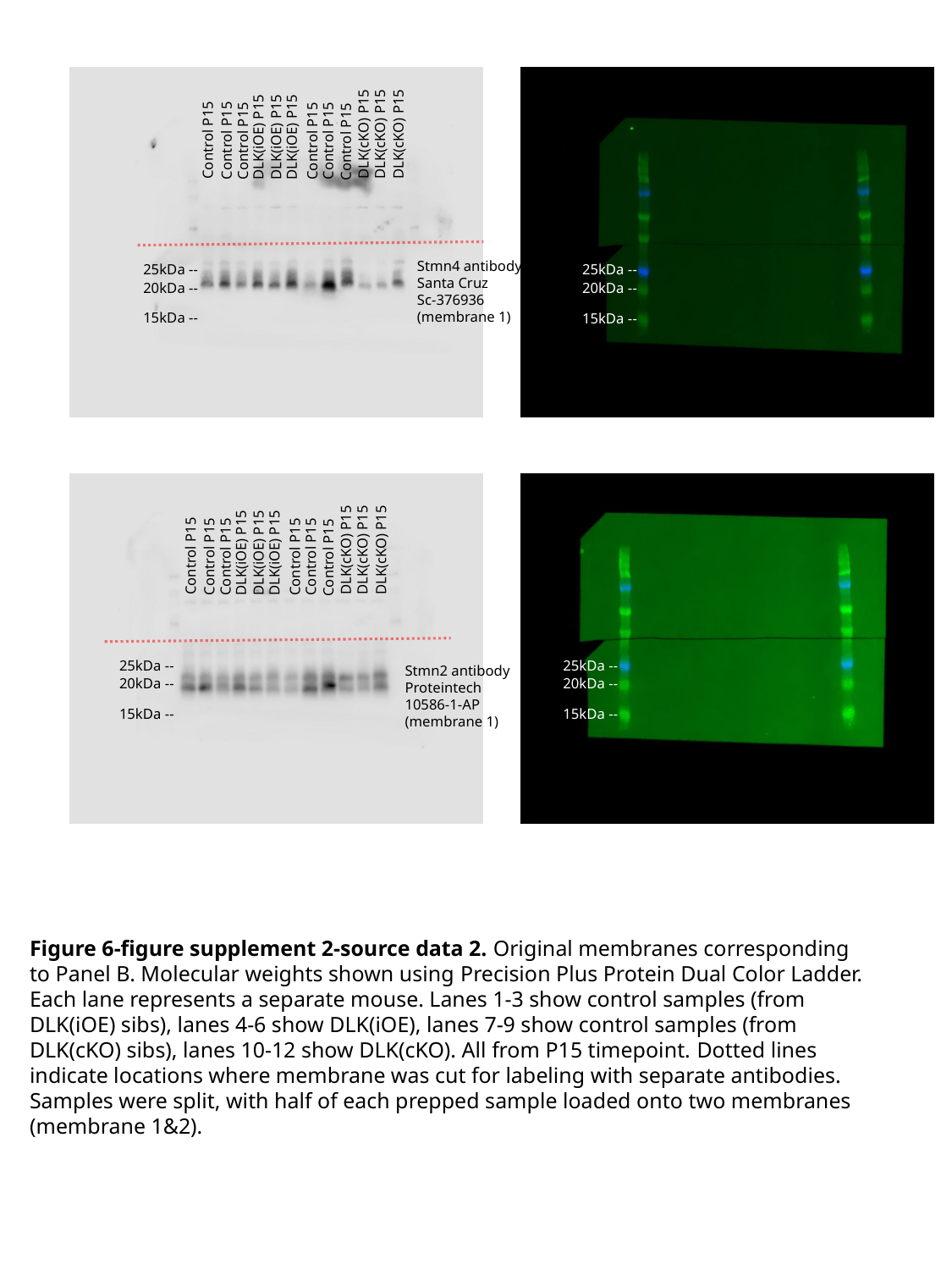

Control P15
DLK(cKO) P15
DLK(cKO) P15
DLK(cKO) P15
Control P15
Control P15
Control P15
Control P15
DLK(iOE) P15
DLK(iOE) P15
DLK(iOE) P15
Control P15
Stmn4 antibody
Santa Cruz
Sc-376936
(membrane 1)
25kDa --
25kDa --
20kDa --
20kDa --
15kDa --
15kDa --
Control P15
DLK(cKO) P15
DLK(cKO) P15
DLK(cKO) P15
Control P15
Control P15
Control P15
Control P15
DLK(iOE) P15
DLK(iOE) P15
DLK(iOE) P15
Control P15
25kDa --
25kDa --
Stmn2 antibody
Proteintech
10586-1-AP
(membrane 1)
20kDa --
20kDa --
15kDa --
15kDa --
Figure 6-figure supplement 2-source data 2. Original membranes corresponding to Panel B. Molecular weights shown using Precision Plus Protein Dual Color Ladder. Each lane represents a separate mouse. Lanes 1-3 show control samples (from DLK(iOE) sibs), lanes 4-6 show DLK(iOE), lanes 7-9 show control samples (from DLK(cKO) sibs), lanes 10-12 show DLK(cKO). All from P15 timepoint. Dotted lines indicate locations where membrane was cut for labeling with separate antibodies. Samples were split, with half of each prepped sample loaded onto two membranes (membrane 1&2).

## Slide 2
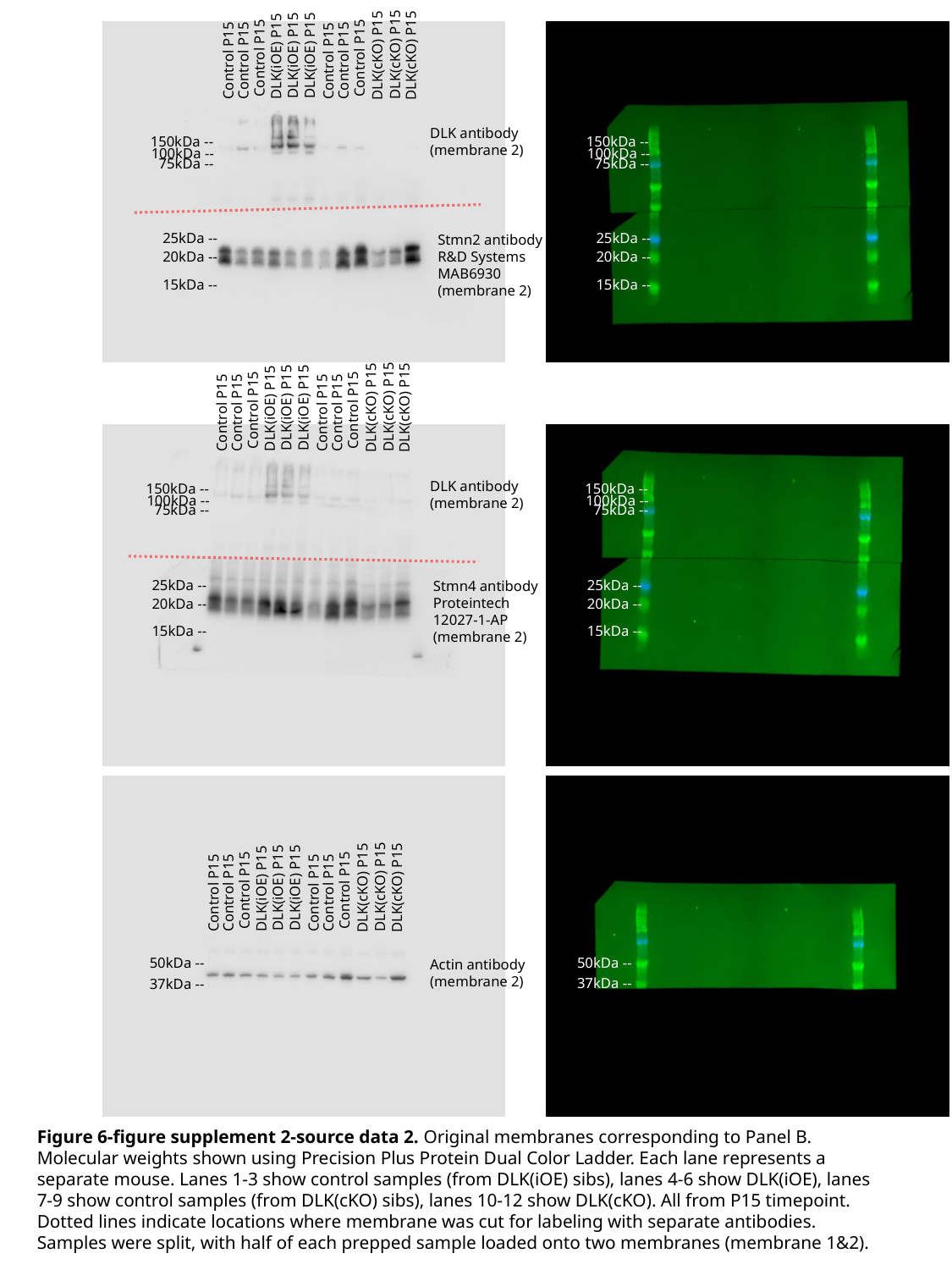

Control P15
Control P15
DLK(iOE) P15
DLK(iOE) P15
Control P15
DLK(iOE) P15
Control P15
DLK(cKO) P15
Control P15
Control P15
DLK(cKO) P15
DLK(cKO) P15
DLK antibody
(membrane 2)
150kDa --
150kDa --
100kDa --
100kDa --
75kDa --
75kDa --
25kDa --
25kDa --
Stmn2 antibody
R&D Systems
MAB6930
(membrane 2)
20kDa --
20kDa --
15kDa --
15kDa --
Control P15
Control P15
DLK(iOE) P15
DLK(iOE) P15
Control P15
DLK(iOE) P15
Control P15
DLK(cKO) P15
Control P15
Control P15
DLK(cKO) P15
DLK(cKO) P15
DLK antibody
(membrane 2)
150kDa --
150kDa --
100kDa --
100kDa --
75kDa --
75kDa --
25kDa --
25kDa --
Stmn4 antibody
Proteintech
12027-1-AP
(membrane 2)
20kDa --
20kDa --
15kDa --
15kDa --
Control P15
Control P15
DLK(iOE) P15
DLK(iOE) P15
Control P15
DLK(iOE) P15
Control P15
DLK(cKO) P15
Control P15
Control P15
DLK(cKO) P15
DLK(cKO) P15
50kDa --
50kDa --
Actin antibody
(membrane 2)
37kDa --
37kDa --
Figure 6-figure supplement 2-source data 2. Original membranes corresponding to Panel B. Molecular weights shown using Precision Plus Protein Dual Color Ladder. Each lane represents a separate mouse. Lanes 1-3 show control samples (from DLK(iOE) sibs), lanes 4-6 show DLK(iOE), lanes 7-9 show control samples (from DLK(cKO) sibs), lanes 10-12 show DLK(cKO). All from P15 timepoint. Dotted lines indicate locations where membrane was cut for labeling with separate antibodies. Samples were split, with half of each prepped sample loaded onto two membranes (membrane 1&2).
