# Supplementary material for: Translatome analysis reveals cellular network in DLK-dependent hippocampal glutamatergic neuron degeneration
Source: eLife. 2025 Mar 11;13:RP101173. doi: 10.7554/eLife.101173 (PMC11896613; doi:10.7554/eLife.101173)
Supplement: Supplementary file 4. — File containing primer sequences used for genotyping and qRT-PCR. [file elife-101173-supp4.docx]

**Supplementary File 4. Primers**

| Primers for genotyping | | | |
| --- | --- | --- | --- |
| Reagent or resource | Source | Identifier | Additional information |
| Hipp11 WT-Fw: TGGAGGAGGACAAACTGGTCA | Li et al., 2021 | YJ12520 | 323bp (*Dlk* Wild-type)  ~500bp (*Dlk^OE^* mutant) |
| Hipp11 WT-Re: TTCCCTTTCTGCTTCATCTTGC | Li et al., 2021 | YJ12521 |  |
| CAG-Re: CATATATGGGCTATGAACTAATGACCCCGT | Li et al., 2021 | YJ12522 |  |
| Map3k12^fl/fl^-Fw: GATGATTGCTAGTCATGGAGTAGTAGG | Li et al., 2021 | YJ12523 | 350bp (*Dlk* Wild-type)  500bp (*Dlk^fl/fl^* mutant) |
| Map3k12^fl/fl^-Re: GGTGGTGTTATCATAGTTCCATCATG | Li et al., 2021 | YJ12524 |  |
| RiboTag Fw: GGGAGGCTTGCTGGATATG | Sanz et al., 2009 |  | 260bp (WT)  290bp (floxed allele) |
| RiboTag Re: TTTCCAGACACAGGCTAAGTA | Sanz et al., 2009 |  |  |
| Slc17a7-cre Re:  CCCTAGGAATGCTCGTCA AG | The Jackson Laboratory | 12231 | 218bp (WT)  344bp (mutant) |
| Slc17a7-cre Fw: ATGAGCGAGGAGAAGTGTGG | The Jackson Laboratory | 17904 |  |
| Slc17a7-cre Re: GTGGAAGTCCTGGAAACTGC | The Jackson Laboratory | 17905 |  |
| Cre Fw: AGAACCTGAAGATGTTCGCG |  |  | ~330bp (mutant)  No band WT |
| Cre Re: GGCTATACGTAACAGGGTGT |  |  |  |
| Rosa tdTomato Re: GGCATTAAAGCAGCGTATCC | The Jackson Laboratory | oIMR9103 | 196bp (mutant)  297bp (WT) |
| Rosa tdTomato Fw: CTGTTCCTGTACGGCATGG | The Jackson Laboratory | oIMR9105 |  |
| WT tdTomato Fw: AAGGGAGCTGCAGRGGAGTA | The Jackson Laboratory | oIMR9020 |  |
| WT tdTomato Re: CCGAAAATGTGTGGGAAGTC | The Jackson Laboratory | oIMR9021 |  |
| Primers for qRT-PCR | | | |
| Reagent or resource | Source | | |
| Gapdh-Fw: GCTTGTCATCAACGGGAAG | Furlanis et al., 2019 | | |
| Gapdh-Re: TTGTCATATTTCTCGTGGTTCA | Furlanis et al., 2019 | | |
| Vgat-Fw: CGTGACAAATGCCATTCAG | Furlanis et al., 2019 | | |
| Vgat-Re: AAGATGATGAGGAACAACCC | Furlanis et al., 2019 | | |
| Slc17a7-Fw: ACCCTGTTACGAAGTTTAACAC | Furlanis et al., 2019 | | |
| Slc17a7-Re: CAGGTAGAAGGTCCAGCTG | Furlanis et al., 2019 | | |
| Wsf1-Fw: CATCATTCCCACCAACCTG | Furlanis et al., 2019 | | |
| Wsf1-Re: TACTTCACCACCTTCTGGC | Furlanis et al., 2019 | | |
| Gfap-Fw: CTCGTGTGGATTTGGAGAG | Furlanis et al., 2019 | | |
| Gfap-Re: AGTTCTCGAACTTCCTCCT | Furlanis et al., 2019 | | |
